# Prognostic and immunological role of Fam20C in pan-cancer

**DOI:** 10.1042/BSR20201920

**Published:** 2021-01-05

**Authors:** Xinpeng Liu, Yuanbo Zhan, Wenxia Xu, Xiaoyao Liu, Yawei Geng, Lixue Liu, Junlong Da, Jianqun Wang, Xinjian Zhang, Han jin, Zhongshuang Liu, Shouli Guo, Bin Zhang, Ying Li

**Affiliations:** 1 Institute of Hard Tissue Development and Regeneration, The Second Affiliated Hospital of Harbin Medical University, Harbin, China; 2 Department of Periodontology and Oral Mucosa, The Second Affiliated Hospital of Harbin Medical University, Harbin, China; 3 Animal Experiment Center of the Second Affiliated Hospital, Harbin Medical University, Harbin, China; 4 Heilongjiang Academy of Medical Sciences, Harbin, China

**Keywords:** database, Fam20C, immune infiltration, pan-cancer, survival analysis

## Abstract

**Background:** The family with sequence similarity 20-member C (Fam20C) kinase plays important roles in physiopathological process and is responsible for majority of the secreted phosphoproteome, including substrates associated with tumor cell migration. However, it remains unclear whether Fam20C plays a role in cancers. Here, we aimed to analyze the expression and prognostic value of Fam20C in pan-cancer and to gain insights into the association between Fam20C and immune infiltration.

**Methods:** We analyzed Fam20C expression patterns and the associations between Fam20C expression levels and prognosis in pan-cancer via the ONCOMINE, TIMER (Tumor Immune Estimation Resource), PrognoScan, GEPIA (Gene Expression Profiling Interactive Analysis), and Kaplan–Meier Plotter databases. After that, GEPIA and TIMER databases were applied to investigate the relations between Fam20C expression and immune infiltration across different cancer types, especially BLCA (bladder urothelial carcinoma), LGG (brain lower grade glioma), and STAD (stomach adenocarcinoma).

**Results:** Compared with adjacent normal tissues, Fam20C was widely expressed across many cancers. In general, Fam20C showed a detrimental role in pan-cancer, it was positively associated with poor survival of BLCA, LGG, and STAD patients. Specifically, based on TCGA (The Cancer Genome Atlas) database, a high expression level of Fam20C was associated with worse prognostic value in stages T2–T4 and stages N0–N2 in the cohort of STAD patients. Moreover, Fam20C expression had positive associations with immune infiltration, including CD4^+^ T cells, macrophages, neutrophils, and dendritic cells, and other diverse immune cells in BLCA, LGG, and STAD.

**Conclusion:** Fam20C may serve as a promising prognostic biomarker in pan-cancer and has positive associations with immune infiltrates.

## Introduction

Protein kinases are a common way of regulating information transduction in organisms, which play a crucial role in the process of cell signal by transferring a phosphate from adenosine triphosphate (ATP) to the target proteins [1,2]. It never really came as a surprise that, protein phosphorylation is an important mechanism involved in multiple physiological processes within the cell [3,4]. What perhaps unexpected was the extracellular protein phosphorylation with the low concentration of ATP in the extracellular environment; phosphoproteomic studies have shown that more than two-thirds of human serum, plasma, and cerebrospinal fluid contain phosphoproteins [5–7]. Emerging evidence has revealed that in physiological functions, extracellular phosphorylation is beneficial for blood coagulation, immune cell activation, and the formation of neuronal networks [8–10]. On the other hand, compelling facts exist that exokinase activity is increased in some diseases, such as cancers [11–13].

Family with sequence similarity 20-member C (Fam20C), as a Golgi casein kinase, can phosphorylate multiple proteins in the secretory pathway within Ser-X-Glu/pSer motif [14–16]. And, there is an evidence display that Fam20C contributes to phosphorylate the most secreted proteins [16]. In addition, with respect to the mutations of Fam20C gene lead to Raine syndrome, which is characterized by generalized osteosclerosis, periosteal bone formation, and a unique facial phenotype [17–19]. Intriguingly, analyzing GO Term of Fam20C substrates shows that Fam20C regulates some biological processes, for instance wound healing, endopeptidase inhibitor activity, lipid homeostasis, cell adhesion and migration in HepG2 cells [16]. Furthermore, the Fam20C substrates were also found to be involved in tumor growth and metastasis, including the insulin-like growth factor binding proteins (IGFBPs), osteopontin (OPN), serine protease inhibitors (Serpins), and several extracellular proteases [20–22]. With respect to cancer progression, only two reports suggest that Fam20C may be a possible therapeutic target for breast cancer (BC) and lung adenocarcinoma (LUAD) [23,24]. However, exploration of Fam20C in cancer has only entered the infant stage. Therefore, in this research, we aim to study Fam20C in pan-cancer in order to draw an outline of the role of Fam20C in tumors.

Tumorigenesis is a complicated process, which is accompanied by enhanced proliferation, resistance to apoptosis, enhanced angiogenesis, escape from immunity and so on [25]. Among them, TME (the tumor microenvironment) plays a critical role. TME consists of cellular components and non-cellular extracellular matrix, cellular components include stromal fibroblasts, infiltrating immune cells, the blood and lymphatic vascular networks [26]. TILs (tumor-infiltrating lymphocytes) play a dual role in cancer, not only suppress tumor growth but also protect cancer cells to escape being killed [27–30]. Therefore, it is necessary to explore the characteristics of various immune cells and the mechanism of their interaction with tumors.

In the present study, we comprehensively analyzed the expression of Fam20C and its correlation with prognostic value in pan-cancer via different databases, including Oncomine, TIMER (Tumor Immune Estimation Resource), PrognoScan, and Kaplan–Meier plotter. In addition, the association between Fam20C and immune infiltration degree was also analyzed using the TIMER and GEPIA (Gene Expression Profiling Interactive Analysis) databases. We observed that Fam20C widely expressed across many cancers and may affect the prognosis of patients by interacting with infiltrating immune cells.

## Materials and methods

### Oncomine database analysis

Fam20C mRNA expression levels in different cancer types were compared with their matched paracancer tissues by using online Oncomine analysis tools. Oncomine (https://www.oncomine.org/) is a major cancer microarray repository and web-based data-mining platform [31,32], which contains 715 datasets and 86733 samples. Data retrieval, analysis, visualization for biomedical research can be evaluated by accessing Oncomine. In our experiment, the parameters were set as follows: *P*-value <0.001, Fold change > 2.

### PrognoScan database analysis

With the intent to explore prognostic condition of Fam20C research, we utilized PrognoScan (http://dna00.bio.kyutech.ac.jp/PrognoScan/index.html), a freely available resource investigating survival information of individual genes among multiple cancers. The clinically annotated cancer microarray datasets were collected from GEO (Gene Expression Omnibus), ArrayExpress, and individual laboratory websites for 14 cancer types, then applied minimum *P*-value approach to analyze [33,34]. For the assessment of prognostic value, some common end points were employed such as OS (overall survival), RFS (recurrence/relapse-free survival), EFS (event-free survival), and distant metastasis-free survival (DMFS).

### Kaplan–Meier plotter database analysis

We subsequently expanded our prognosis-related investigation to include the Kaplan–Meier Plotter database (https://kmplot.com/analysis/), which is an online database including gene expression data and clinical data, commonly used for assessing different genes on survival among 21 cancer types [35]. Moreover, this database currently contains 6234 breast, 2190 ovarian, 3452 lung, and 1440 gastric cancer datasets. Importantly, the data sources of this database include not only GEO, but also European Genome-phenome Archive (EGA) and TCGA (The Cancer Genome Atlas), which are not covered by PrognoScan Database. The prognostic significance of the mRNA expression of Fam20C in different 21 human cancers was evaluated using the Kaplan–Meier plotter database, the hazard ratio with 95% confidence intervals and log rank *P*-value were also obtained.

### GEPIA

Both the RNA sequencing expression data of 9736 tumors and 8587 normal samples from the TCGA and GTEx (Genotype-Tissue Expression) projects can be obtained by accessing the GEPIA (http://gepia.cancer-pku.cn/index.html) web server, as a building block in an interactive and customizable resource for research. Functions thus far include differential expression analysis, survival analysis, correlation analysis, profiling plotting, similar gene detection, and dimensionality reduction analysis [36]. Remarkably, this interactive web server includes 33 malignant tumors for users to explore interested information. In the present study, the GEPIA database was used to verify the relevant results obtained from the application of the Oncomine database, and then ‘Survival Plots’ module was applied to analyze the survival prognosis of Fam20C. Further, through the ‘Correlation Analysis’ module, we explored the relationship between the expression of the Fam20C gene and the immune gene markers.

### TIMER database analysis

With respect to tumor immune research, TIMER (https://cistrome.shinyapps.io/timer/) provides a user-friendly web interface to explore and visualize tumor immunologic and genomics data [37]. Information thus far includes 10897 samples of 32 cancers from TCGA, together with the abundance of TIICs (tumor-infiltrating immune cells) based on a deconvolution method from gene expression profiles [38]. In this research, we utilized ‘Gene’ module to estimate the correlation between Fam20C expression and immune infiltration level (the abundance of six TIIC subgroups: B cells, CD4^+^ T cells, CD8^+^ T cells, macrophages, neutrophils, and dendritic cells), as well as tumor purity, among 39 cancer types. And then, the ‘Correlation’ module was applied to analyze the association between Fam20C and other prognosis-related immune cell markers to further estimate the potential infiltrating immune cells subtypes. These gene markers include B cells, CD8^+^ T cells, dendritic cells, exhausted T cells, macrophages, M1 macrophages, M2 macrophages, monocytes, TAMs (tumor-associated macrophages), neutrophils, natural killer (NK) cells, follicular helper T cells (Tfh), Regulatory cells (Tregs), T-helper 1 (Th1) cells, T-helper 2 (Th2) cells, T-helper 9 (Th9) cells, T-helper 17 (Th17) cells, and T-helper 22 (Th22) cells. Moreover, we selected the immune gene markers by searching the website of CellMarker (http://biocc.hrbmu.edu.cn/CellMarker/). The expression level of gene was adjusted by log2 TPM (transcripts per million). Fam20C was plotted on the x-axis, while marker genes were plotted on the y-axis. The expression scatterplots can visualize correlations between Fam20C and each immune gene marker.

### Statistical analysis

Correlation datasets for the differential expression of cancer and adjacent tissues were created in Oncomine with *P*-values, fold changes, and gene ranks. Survival curves were drawn by the PrognoScan, Kaplan–Meier plotter, and GEPIA. The hazard ratio and Cox *P*-values or log-rank *P*-values were used for comparing OS, RFS, EFS, and DMFS among patients in different groups. The correlation of gene expression analyzed in GEPIA and TIMER, in which Spearman’s correlation was employed as correlation coefficient. Throughout the text, a *P*-value <0.05 was examined to be statistically significant.

## Results

### Fam20C mRNA expression levels across different cancers

**Figure 1:**
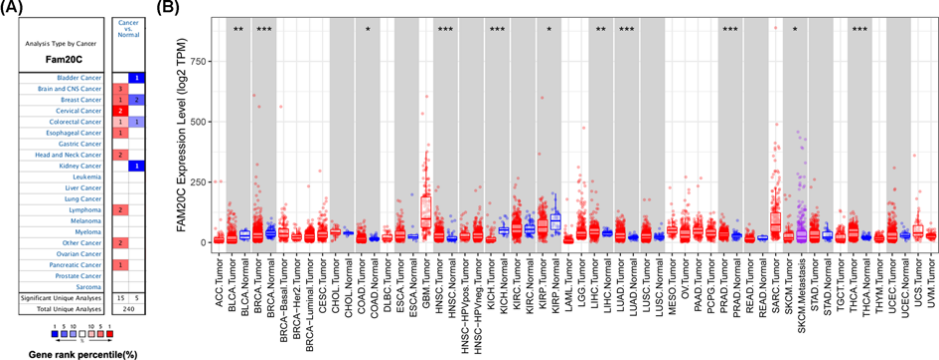
Fam20C expression levels in different cancer types (**A**) Increased or decreased expression of Fam20C compared with normal tissues across different cancer types in Oncomine database. The number in each cell present the amount of datasets. (**B**) Human Fam20C expression levels across different cancer types from TCGA database in TIMER (*P<0.05, **P<0.01, ***P<0.001).

The expression levels of Fam20C mRNA across different cancers, between tumor and normal tissue, were analyzed in Oncomine and TIMER databases. In Oncomine database, compared with the normal tissues the result revealed higher expression of Fam20C in brain and CNS (central nervous system), breast, cervical, esophageal, head and neck, lymphoma, and pancreatic tumors (Figure 1A). In contrast, decreased expression of Fam20C was found in bladder and kidney cancers. Notably, elevated Fam20C expression was demonstrated in only one BC dataset, but two decreased expression were observed in two BC datasets. Similarly, in colorectal cancer, compared with normal tissue, there is one dataset with higher expression and one with lower expression. Detailed results of Fam20C expression across different cancer types are summarized in Supplementary Table S1.

To further verify the expression levels of Fam20C in cancerous and normal tissues across all TCGA tumors, we next profiled and compared them in TIMER platform. Specifically, Fam20C expression levels were significantly elevated in HNSC (head and neck squamous carcinoma), LIHC (liver hepatocellular carcinoma), LUAD, PRAD (prostate adenocarcinoma), and THCA (thyroid carcinoma) (Figure 1B). Moreover, the decreased expression of Fam20C was found in BLCA (bladder urothelial carcinoma), BRCA (breast invasive carcinoma), COAD (colon adenocarcinoma), KICH (kidney chromophobe), KIRP (kidney renal papillary cell carcinoma), LIHC, and SKCM (skin cutaneous melanoma).

### Fam20C as a potential oncogene, prognosis in different cancers

**Figure 2:**
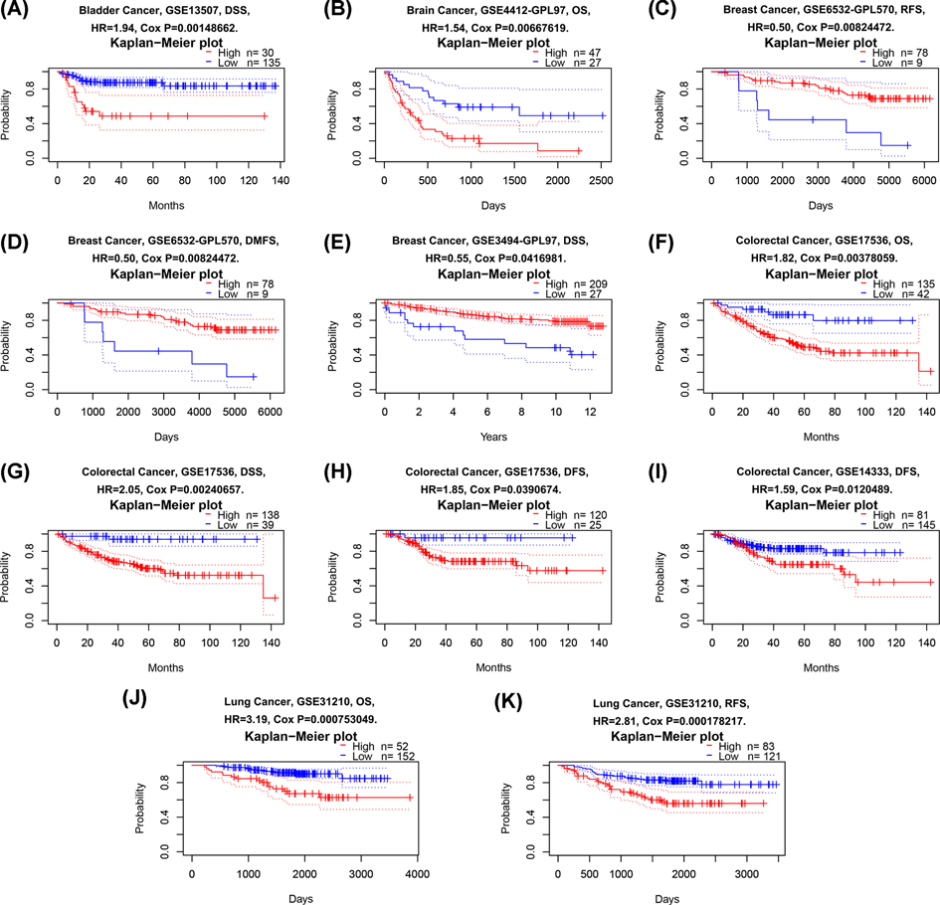
Kaplan–Meier survival curves comparing high and low expression of Fam20C in different cancer types in PrognoScan (**A**) DSS (*n*=165) in bladder cancer cohort GSE13507. (**B**) OS (*n*=74) in brain cancer cohort GSE4412-GPL97. (**C,D**) RFS (*n*=87) and DMFS (*n*=87) in BC cohort GSE6532-GPL570. (**E**) DSS (*n*=236) and in BC cohort GSE3494-GPL97. (**F**–**H**) OS (*n*=177), DSS (*n*=177) and DFS (*n*=145) in colorectal cancer cohort GSE17536. (**I**) DFS (*n*=226) in colorectal cancer cohort GSE14333. (**J,K**) OS (*n*=204) and RFS (*n*=204) in lung cancer cohort GSE31210.

Although previous studies have reported that expression of Fam20C was related to two cancer types (BRCA and LUAD) [23,24], the prognostic values of Fam20C has not been given enough attention. In PrognoScan database (data source from GEO), we performed an analysis to identify the cancer types which were related to Fam20C expression. The results showed that four cancer types displayed poorer prognosis, including bladder [DSS (disease-specific survival): total number, 165; 95% CI, 1.29–2.92; HR, 1.94; Cox *P*, 0.00148662], brain [OS: total number, 74; 95% CI, 1.13–2.10; HR, 1.54; Cox *P*, 0.00667619], colorectal [OS: total number, 177; 95% CI, 1.21–2.72; HR, 1.82; Cox *P*, 0.00378059; DSS: total number, 177; 95% CI, 1.29–3.26; HR, 2.05; Cox *P*, 0.00240657; DFS (disease-free survival): total number, 145; 95% CI, 1.03–3.32; HR, 1.85; Cox *P*, 0.0390674; another dataset showed DFS: total number, 226; 95% CI, 1.11–2.29; HR, 1.59; Cox *P*, 0.0120489], and lung cancers [OS: total number, 204; 95% CI, 1.63–6.28; HR, 3.19; Cox *P*, 0.000753049; RFS: total number, 204; 95% CI, 1.64–4.82; HR, 2.81; Cox *P*, 0.000178217] (Figure 2A,B,F–K and Supplementary Figure S1). In addition, there is an association between higher Fam20C expression and better prognosis in the BC [RFS: total number, 87; 95% CI, 0.29–0.83; HR, 0.5; Cox *P*, 0.00824472; DMFS: total number, 87; 95% CI, 0.29–0.83; HR, 0.5; Cox *P*, 0.00824472; DSS: total number, 236; 95% CI, 0.31–0.98; HR, 0.55; Cox *P*, 0.0416981) (Figure 2C–E).

Likewise, the same work was performed in Kaplan–Meier plotter database (data source from TCGA), OS and RFS were used as indicators to judge the prognostic value, seven cancer types, respectively, exhibited bad prognosis and good prognosis on mRNA abundance of Fam20C.

**Figure 3:**
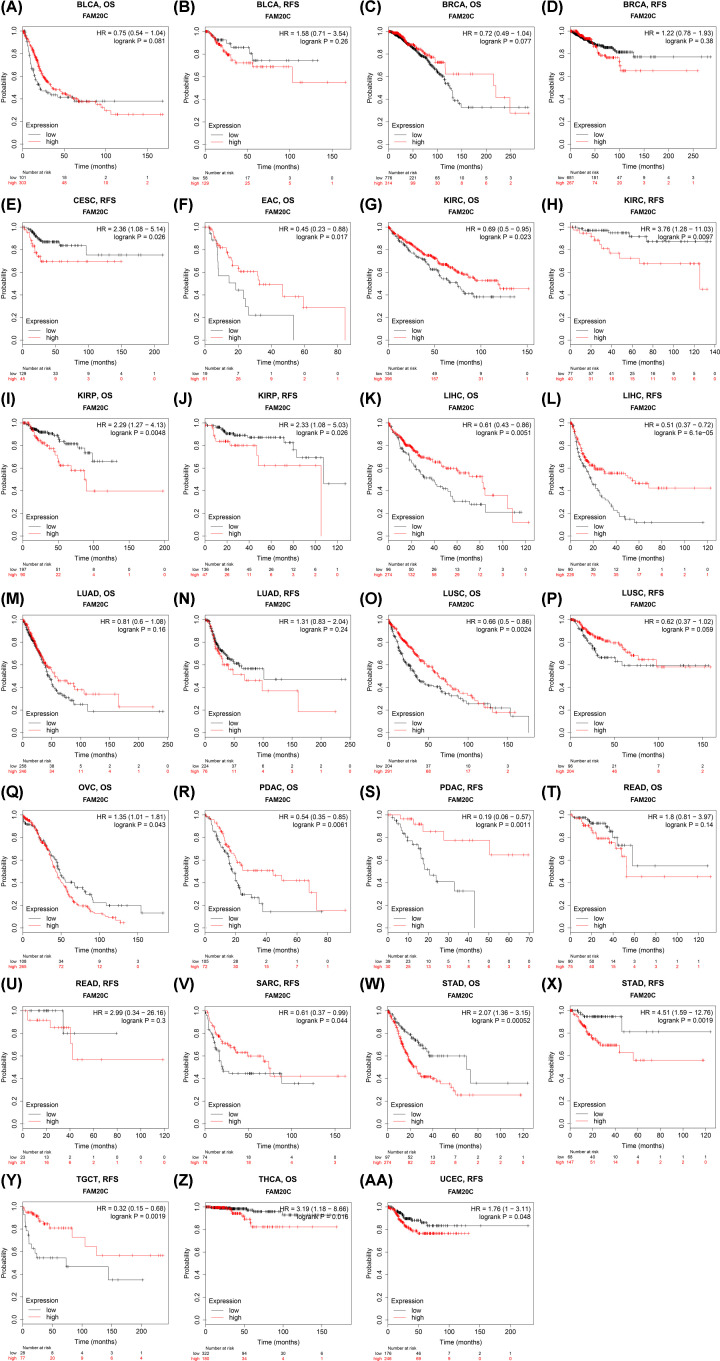
Kaplan–Meier survival curves comparing the high and low expression of Fam20C in different types of cancer in Kaplan–Meier Plotter (**A,B**) OS and RFS in BLCA. (**C,D**) OS and RFS in breast cancer (BRCA). (**E**) RFS in CESC. (**F**) OS in EAC. (**G,H**) OS and RFS in kidney renal clear cell carcinoma (KIRC). (**I,J**) OS and RFS in KIRP. (**K,L**) OS and RFS in LIHC. (**M,N**) OS and RFS in LUAD. (**O,P**) OS and RFS in lung squamous cell carcinoma (LUSC). (**Q**) OS in ovarian cancer (OVC). (**R,S**) OS and RFS in pancreatic ductal adenocarcinoma (PDAC). (**T,U**) OS and RFS in READ. (**V**) RFS in sarcoma (SARC). (**W,X**) OS and RFS in STAD. (**Y**) RFS in TGCT. (**Z**) OS in THCA. (**AA**) RFS in UCEC.

Especially among them, Fam20C worsened RFS in kidney renal clear cell carcinoma but benefited OS (KIRC: OS: HR, 0.69; 95% CI, 0.5–0.95; logrank *P*, 0.023; RFS: HR, 3.76; 95% CI, 1.28–11.03; logrank *P*, 0.0097) (Figure 3G,H). The results in Kaplan–Meier plotter database were extremely different from that using PrognoScan, these signatures (OS and RFS) showed no significant association with bladder carcinoma (BLCA: OS: HR, 0.75; 95% CI, 0.54–1.04; logrank *P*, 0.081; RFS: HR, 1.58; 95%CI, 0.71-3.54; logrank *P*, 0.26) (Figure 3A,B), breast cancer (BRCA: OS: HR, 0.72; 95% CI, 0.49–1.04; logrank *P*, 0.077; RFS: HR, 1.22; 95% CI, 0.78-1.93; logrank *P*, 0.38) (Figure 3C,D), and rectum adenocarcinoma (READ: OS: HR, 1.8; 95% CI, 0.81–3.97; logrank *P*, 0.14; RFS: HR, 2.99; 95% CI, 0.34–26.16; logrank *P*, 0.3) (Figure 3T,U). In contrast with PrognoScan, Fam20C expression up-regulation benefited OS of lung squamous cell carcinoma in lung cancers (LUAD: OS: HR, 0.81; 95% CI, 0.6–1.08; logrank *P*, 0.16; RFS: HR, 1.31; 95% CI, 0.83–2.04; logrank *P*, 0.24; LUSC: OS: HR, 0.66; 95% CI, 0.5–0.86; logrank *P*, 0.0024; RFS: HR, 0.62; 95% CI, 0.37–1.02; logrank *P*, 0.059) (Figure 3M–P). Exceptionally, it does not analyze about brain cancer in Kaplan–Meier plotter. In addition, the worse prognostic role of Fam20C was observed in six cancer types, including cervical squamous cell carcinoma (CESC: RFS: HR, 2.36; 95% CI, 1.08–5.14; logrank *P*, 0.026) (Figure 3E), KIRP (OS: HR, 2.29; 95% CI, 1.27–4.13; logrank *P*, 0.0048; RFS: HR, 2.33; 95% CI, 1.08–5.03; logrank *P*, 0.026) (Figure 3I,J), ovarian cancer (OVC: OS: HR, 1.35; 95% CI, 1.01–1.81; logrank *P*, 0.043) (Figure 3Q), STAD (stomach adenocarcinoma: OS: HR, 2.07; 95% CI, 1.36–3.15; logrank *P*, 0.00052; RFS: HR, 4.51; 95% CI, 1.59–12.76; logrank *P*, 0.0019) (Figure 3W,X), THCA (OS: HR, 3.19; 95% CI, 1.18–8.66; logrank *P*, 0.016) (Figure 3Z), UCEC (uterine corpus endometrial carcinoma: RFS: HR, 1.76; 95% CI, 1–3.11; logrank *P*, 0.048) (Figure 3AA). Further, Fam20C played a protective role in some cancer types, including esophageal adenocarcinoma (EAC: OS: HR, 0.45; 95% CI, 0.23–0.88; logrank *P*, 0.017) (Figure 3F), LIHC (OS: HR, 0.61; 95% CI, 0.43–0.86; logrank *P*, 0.0051; RFS: HR, 0.51; 95% CI, 0.37–0.72; logrank *P*, 6.1e-05) (Figure 3K,L), pancreatic ductal adenocarcinoma (PDAC: OS: HR, 0.54; 95% CI, 0.35–0.85; logrank *P*, 0.0061; RFS: HR, 0.19, 95% CI, 0.06–0.57; logrank *P*, 0.0011) (Figure 3R,S), sarcoma (SARC: RFS: HR, 0.61; 95% CI, 0.37–0.99; logrank *P*, 0.044) (Figure 3V), and testicular germ cell tumor (TGCT: RFS: HR, 0.32; 95% CI, 0.15–0.68; logrank *P*, 0.0019) (Figure 3Y).

**Figure 4:**
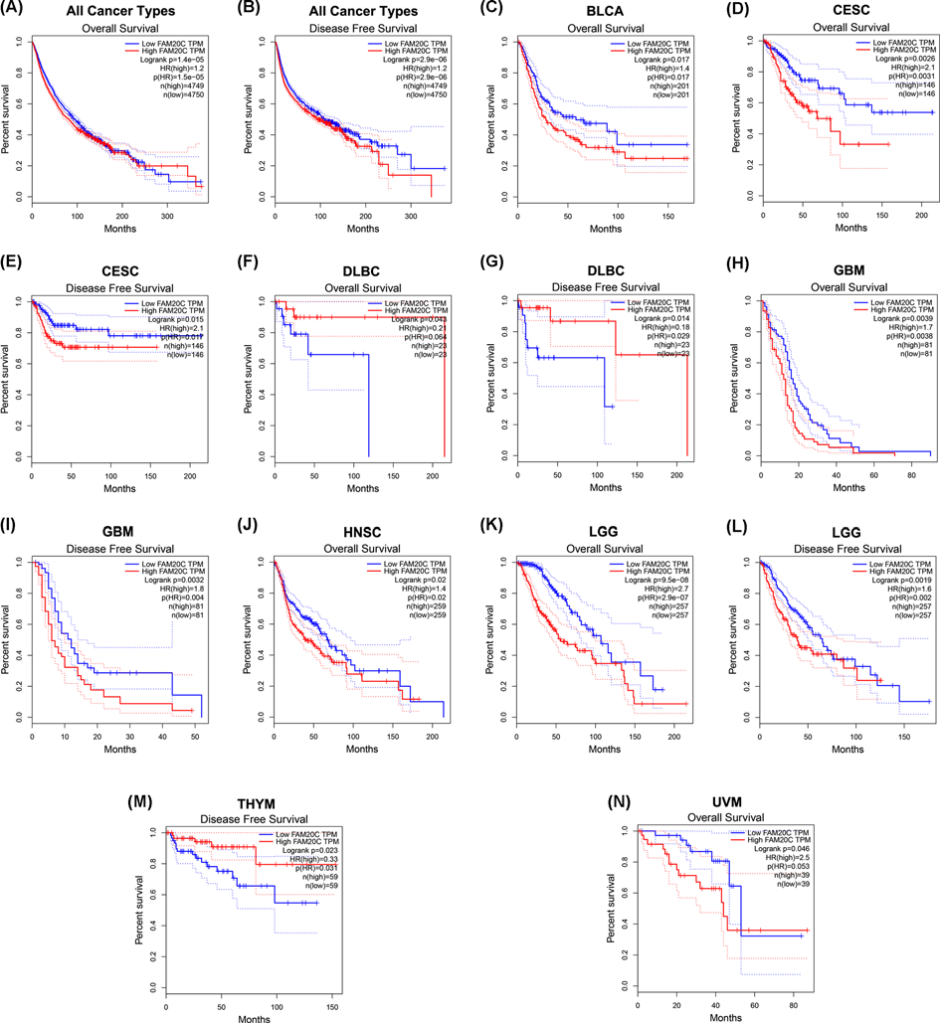
Kaplan–Meier survival curves comparing the high and low expression of Fam20C across different types of cancer in GEPIA (**A,B**) OS and DFS in all cancer types. (**C**) OS in bladder carcinoma (BLCA). (**D,E**) OS and DFS in CESC. (**F,G**) OS and DFS in lymphoid neoplasm diffuse large B-cell lymphoma (DLBC). (**H,I**) OS and DFS in glioblastoma multiforme (GBM). (**J**) OS in HNSC. (**K,L**) OS and DFS in brain lower grade glioma (LGG). (**M**) DFS in thymoma (THYM). (**N**) OS in UVM (uveal melanoma).

To further clarify its role of FAM20C in pan-cancer, the GEPIA database, which can provide more cancer types was used. Similarly, higher mRNA levels of Fam20C gave a worse prognostic prediction in all cancers (OS: total number, 9499; HR, 1.2; logrank *P*, 1.4e-05; DFS: total number, 9499; HR, 1.2; logrank *P*, 2.9e-06) (Figure 4A,B).

Consistent with the results from PrognoScan and Kaplan–Meier plotter database, high expression of Fam20C had a poorer prognosis roles in 33 kinds of cancer types data that were retrieved from GEPIA, including BLCA (OS: total number, 402; HR, 1.4; logrank *P*, 0.017) (Figure 4C and Supplementary Figure S2), CESC (OS: total number, 292; HR, 2.1; logrank *P*, 0.0026; DFS: total number, 292; HR, 2.1; logrank *P*, 0.015) (Figure 4D,E), and brain cancer includes glioblastoma multiforme (GBM: OS: total number, 162; HR, 1.7; logrank *P*, 0.0039; DFS: total number, 162; HR, 1.8; logrank *P*, 0.0032) (Figure 4H,I) and brain lower grade glioma (LGG: OS: total number, 514; HR, 2.7; logrank *P*, 9.5e-08; DFS: total number, 514; HR, 1.6; logrank *P*, 0.0019) (Figure 4K,L) Moreover, we observed the association of both HNSC (OS: total number, 518; HR, 1.4; logrank *P*, 0.02) (Figure 4J), and UVM (uveal melanoma: OS: total number, 78; HR, 2.5; logrank *P*, 0.046) (Figure 4N).

On the contrary, Fam20C played a positive role in prognostic value in lymphoid neoplasm diffuse large B-cell lymphoma (DLBC: OS: total number, 46; HR, 0.21; logrank *P*, 0.043; DFS: total number, 46; HR, 0.18; logrank *P*, 0.014) (Figure 4F,G), and thymoma (THYM: DFS: total number, 118; HR, 0.33; logrank *P*, 0.023) (Figure 4M).

Together, these integrated analyses confirmed that Fam20C had prognostic value in certain cancers, which may be protective or harmful. In general, the expression of Fam20C showed a detrimental role in pan-cancer.

### Association of lymphatic metastasis in STAD patients with high Fam20C expression

**Figure 5:**
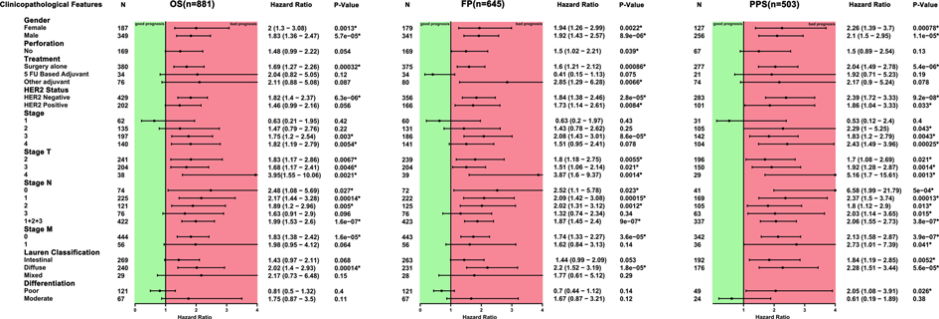
Correlation of Fam20C mRNA expression with OS (*n*=881), FP (*n*=645), and PFS (*n*=503) in STAD with different clinicopathological factors in Kaplan–Meier plotter database Black plots represent hazard ratio, green squares represent good prognosis, and red squares represent bad prognosis. Short bars appear due to limited sample size for parameters. PFS, progression-free survival. *P<0.05.

We next sought to find the relevance and potential mechanisms underlying Fam20C expression in cancers. Thus we analyzed the relationship between the Fam20c expression and several characteristics of gastric cancer patients by using the Kaplan–Meier plotter database, which includes clinical features and pathological stages. Consequently, a strong association of the Fam20C high expression with worse OS, FP (first progression), and PPS (post-progression survival) in female and male patients was found. Interestingly, a similar association was observed both in surgery alone or treatment and HER2 negative (*P*<0.05) (Figure 5). For clinicopathological factors, the association of elevated Fam20C expression with OS, FP and PSS was found in stage 3, PSS in stage 2, OS and PSS in stage 4 of gastric cancer patients. Notably, Fam20C played a detrimental role on local lymph node involvement in OS, FP, and PPS among N0–N3. In addition, Fam20C seemed to only affect gastric cancer patients without distant metastases. The depth of tumor invasion (T category) and the number of positive lymph nodes (N category) had been proved to be two most important prognostic factors [39,40]. These results indicated that up-regulated Fam20C markedly impacted the lymph node metastasis, predicting worse prognosis.

### Fam20C influenced the extent of immune infiltration in BLCA, LGG, and STAD

**Figure 6:**
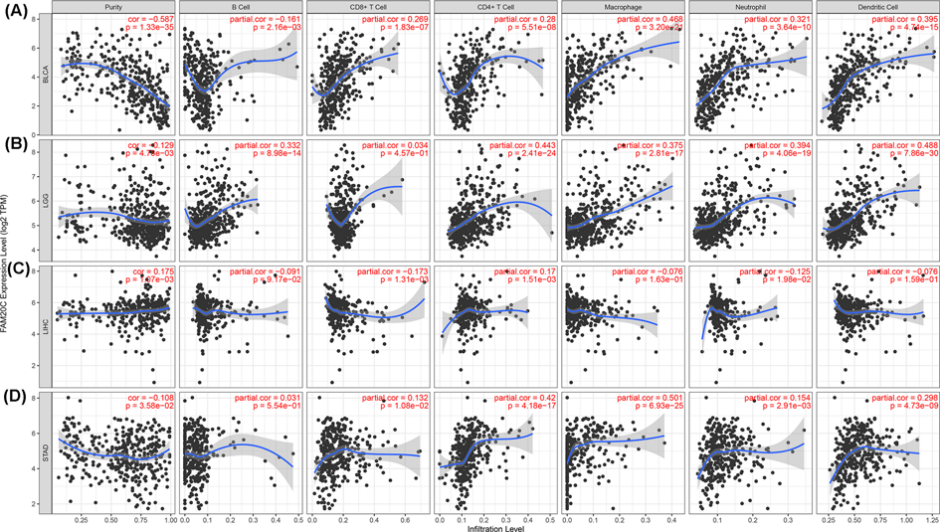
Correlation of Fam20C expression with immune infiltration level in BLCA, LGG, LIHC, and STAD (**A**) Fam20C expression is significantly negatively related to tumor purity has significant positive correlations with infiltrating levels of CD8^+^ T cells, CD4^+^ T cells, macrophages, neutrophils, and dendritic cells in BLCA. Fam20C expression shows a very weak correlation with B-cells infiltration level in BLCA. (**B**) Fam20C expression has a week correlation with tumor purity and is significantly positively correlated with infiltrating levels of B cells, CD4^+^ T cells, macrophages, neutrophils, and dendritic cells in LGG, other than CD8^+^ T cells. (**C**) Fam20C expression is positively related to tumor purity and CD4^+^ T cells, and has a negatively weak relation with CD8^+^ T cells infiltration level in LIHC. Further, there is no relation with infiltrating levels of B cells, macrophages, neutrophils, and dendritic cells. (**D**) Fam20C expression is negatively related to tumor purity and has significant positive correlations with infiltrating levels of CD4^+^ T cells, macrophages, neutrophils, and dendritic cells in STAD but no significant correlation with infiltrating level of B cells and CD8^+^ T cells.

Numerous papers and reviews suggest that multiple types of immune cells are associated with prognosis in various cancer types and of particular importance are the TILs [41–45]. Deeply understanding the immune activity of TILs in cancer would provide more accurate prognostic information. Hence, Spearman’s correlation coefficient was applied to analyze the correlation between Fam20C and immune infiltration level across 39 cancer types in TIMER. This analysis revealed that Fam20C was correlated with decreased purity of tumor in 19 cancer types and increased purity of tumor in two cancers. Furthermore, the association was also observed for 9, 12, 24, 23, 21, and 24 cancer types, corresponding to the B cells, CD8^+^ T cells, CD4^+^ T cells, macrophages, neutrophils, and dendritic cells infiltration levels, respectively (Figure 6 and Supplementary Figure S3).

Considering Fam20C expression correlated with levels of immune invasion in many types of cancers, we next performed the analysis combination of immune infiltration and prognosis. Tumor purity can be interpreted as the proportion of tumor cells in tumor tissue, immune-related genes are negatively correlated with tumor purity regardless of tumor purity [46]. Most of intersection data from TCGA were covered in TIMER and GEPIA databases. Consequently, we selected cancer types in which the elevated Fam20C was negatively related to the tumor purity in TIMER and was largely related to bad prognosis in GEPIA. As noted above, BLCA and LGG were selected, furthermore STAD was also included in this analysis, which was the only one that had poor OS and RFS with high expression of Fam20C in Kaplan–Meier plotter and also had a high level of infiltration in GEPIA. We observed positive correlation between Fam20C expression and infiltrating levels of CD8^+^ T cells (R, 0.269; *P*, 1.83e-07), CD4^+^ T cells (R, 0.28; *P*, 5.51e-08), macrophages (R, 0.468; *P*, 3.20e-21), neutrophils (R, 0.321; *P*, 3.64e-10), and DCs (R, 0.395; *P*, 4.74e-15) in BLCA (Figure 6). For LGG, the results also displayed a positive correlation with infiltrating levels of B cell (R, 0.332; *P*, 8.98e-14), CD4+ T cells (R, 0.443; *P*, 2.41e-24), macrophages (R, 0.375; *P*, 2.81e-17), neutrophils (R, 0.394; *P*, 4.06e-19), and DCs (R, 0.488; *P*, 7.86e-30). Similar to BLCA and LGG, the positive correlation with infiltrating levels of STAD as follows: CD8^+^ T cells (R, 0.132; *P*, 1.08e-02), CD4^+^ T cells (R, 0.42; *P*, 4.18e-17), macrophages (R, 0.501; *P*, 6.93e-25), neutrophils (R, 0.154; *P*, 2.91e-03) and DCs (R, 0.298; *P*, 4.73e-09). Also, tumor purity was negatively correlated with Fam20C expression in BLCA (R, −0.587; *P*, 1.33e-35), LGG (R, −0.129; *P*, 4.78e-03), and STAD (R, −0.108; *P*, 3.58e-02). However, for BLCA and STAD, Fam20C showed no correlation with the CD4^+^ T cells infiltration degree and there was similar condition with CD8^+^ T cells in LGG. Besides, Fam20C expression has no obvious relation with tumor purity and infiltrating levels of B cells, CD8^+^ T cells, CD4^+^ T cells, macrophages, and neutrophils in DLBC, at the same time in GEPIA showed Fam20C played a protective role of prognosis in DLBC. Collectively, these findings may demonstrate that Fam20C could affect the intratumor densities of immune cells.

### Correlation between Fam20C expression and immune markers

Beyond the correlation between Fam20C and the above six immune infiltrating cells, we next sought to find whether Fam20C was associated with the expression of more immune infiltrating cells by investigating related immune cell markers among BLCA, LGG, and STAD in TIMER and GEPIA. Immune cells were recognized by cell markers, including B cells, T cells (general), CD8^+^ T cells, different functional T cells, M1 and M2 macrophages, TAMs, monocytes, NK cells, neutrophils, and dendritic cells in BLCA, LGG, and STAD, using LIHC as the control. After correlating adjustment by purity, we observed that the expression of Fam20C was strongly associated with 60 among 72 immune cell markers in BLCA, 59 in LGG, and 53 in STAD. However, there was significant correlation with only five gene markers in LIHC (Table 1).

**Table 1:** Analysis of the correlation between Fam20C and gene markers of immune cells in TIMER

Cell type	Marker	BLCA				LGG				STAD				LIHC			
		None		Purity		None		Purity		None		Purity		None		Purity	
		Cor	*P*	Cor	*P*	Cor	*P*	Cor	*P*	Cor	*P*	Cor	*P*	Cor	*P*	Cor	*P*
B cell	CD19	0.397	***	0.144	*	0.327	***	0.316	***	0.323	***	0.326	***	−0.099	0.0575	−0.047	0.386
	CD27	0.535	***	0.277	***	0.361	***	0.369	***	0.278	***	0.268	***	−0.144	*	−0.049	0.363
	CD79A	0.481	***	0.23	***	0.201	***	0.222	0.0247	0.318	***	0.311	**	−0.086	0.0974	0.019	0.722
T cell (general)	CD3D	0.448	***	0.18	**	0.44	***	0.429	***	0.144	*	0.125	0.0153	−0.199	**	−0.124	0.0211
	CD3E	0.528	***	0.253	***	0.433	***	0.422	***	0.212	***	0.202	***	−0.169	*	−0.068	0.211
	CD2	0.521	***	0.257	***	0.434	***	0.425	***	0.185	**	0.171	**	−0.163	*	−0.061	0.257
CD8^+^ T cell	CD8A	0.458	***	0.227	***	0.241	***	0.206	***	0.222	***	0.202	***	−0.131	0.0114	−0.037	0.498
	CD8B	0.35	***	0.187	**	0.061	0.169	0.046	0.316	0.105	0.032	0.095	0.0655	−0.13	0.012	−0.035	0.511
Tfh	CXCR3	0.545	***	0.305	***	0.47	***	0.453	***	0.18	**	0.173	**	−0.125	0.0164	−0.038	0.482
	CXCR5	0.479	***	0.207	***	0.235	***	0.235	***	0.37	***	0.361	***	−0.08	0.123	0.007	0.893
	ICOS	0.469	***	0.202	***	0.4	***	0.382	***	0.132	*	0.113	0.0275	−0.207	***	−0.132	0.0143
Th1	IFN-γ (IFNG)	0.358	***	0.166	*	0.256	***	0.246	***	−0.148	*	−0.153	*	−0.138	*	−0.074	0.173
	STAT4	0.534	***	0.293	***	−0.097	0.0271	−0.115	0.0121	0.247	***	0.245	***	−0.163	*	−0.111	0.0391
	STAT1	0.335	***	0.122	0.0195	0.345	***	0.325	***	−0.019	0.703	−0.019	0.715	−0.099	0.0558	−0.054	0.314
	CD94 (KLRD1)	0.525	***	0.311	***	0.295	***	0.285	***	0.159	*	0.149	*	−0.073	0.162	0.003	0.949
	BET (TBX21)	0.5	***	0.266	**	0.446	***	0.436	***	0.199	***	0.195	**	−0.069	0.184	0.027	0.622
Th2	STAT6	−0.282	***	−0.238	***	0.236	***	0.218	***	0.233	***	0.232	***	0.169	*	0.174	*
	CD4	0.583	***	0.36	***	0.392	***	0.386	***	0.339	***	0.329	***	−0.136	*	−0.073	0.175
	GATA-3	−0.451	***	-0.327	***	0.428	***	0.424	***	0.328	***	0.333	***	−0.146	*	−0.045	0.406
	CD184 (CXCR4)	0.572	***	0.357	***	0.306	***	0.296	***	0.371	***	0.352	***	−0.115	0.0271	−0.04	0.457
	CD194 (CCR4)	0.437	***	0.217	***	0.29	***	0.259	***	0.414	***	0.405	***	−0.08	0.123	−0.023	0.673
Th9	TGFBR2	0.362	***	0.211	***	0.215	***	0.193	***	0.405	***	0.379	***	0.098	0.0592	0.114	0.0337
	IRF4	0.503	***	0.211	***	0.044	0.322	0.039	0.399	0.277	***	0.264	***	−0.112	0.0308	−0.018	0.738
	SPI1	0.726	***	0.546	***	0.524	***	0.521	***	0.346	***	0.341	***	−0.144	*	−0.061	0.261
Th17	CD161 (KLRB1)	0.452	***	0.205	***	0.366	***	0.353	***	0.226	***	0.206	***	−0.14	*	−0.04	0.462
	CD121A(IL1R1)	0.395	***	0.265	***	0.183	***	0.161	**	0.659	***	0.645	***	0.152	*	0.161	*
	STAT3	0.33	***	0.172	**	0.408	***	0.386	***	0.42	***	0.41	***	0.004	0.932	0.037	0.497
Th22	CCR10	0.255	***	0.237	***	0.539	***	0.544	***	0.487	***	0.489	***	−0.017	0.745	0.009	0.875
	AHR	-0.345	***	-0.267	**	0.315	***	0.288	***	0.203	***	0.184	**	0.182	**	0.193	**
Treg	CD25 (IL2RA)	0.673	***	0.482	***	0.208	***	0.222	***	0.209	***	0.187	**	−0.182	**	−0.11	0.0409
	CCR8	0.432	***	0.216	***	0.189	***	0.209	***	0.299	***	0.281	***	−0.148	*	−0.084	0.119
	FOXP3	0.498	***	0.296	***	0.033	0.448	0.059	0.201	0.254	***	0.231	***	−0.091	0.0815	−0.065	0.23
	CD127 (IL7R)	0.583	***	0.34	***	0.224	***	0.194	***	0.362	***	0.354	***	−0.099	0.0555	−0.012	0.821
Exhausted T cell	PD-1 (PDCD1)	0.514	***	0.286	***	0.508	***	0.485	***	0.21	***	0.206	***	−0.083	0.109	−0.011	0.836
	Tim-3 (HAVCR2)	0.697	***	0.508	***	0.449	***	0.446	***	0.261	***	0.246	***	−0.206	***	−0.128	0.0176
	CTLA4	0.509	***	0.275	***	0.272	***	0.254	***	0.076	0.122	0.061	0.238	−0.244	***	−0.178	**
	LAG3	0.517	***	0.314	***	0.332	***	0.344	***	0.09	0.0666	0.079	0.125	−0.115	0.0262	−0.052	0.339
	GZMB	0.531	***	0.305	***	0.28	***	0.286	***	−0.047	0.338	−0.081	0.115	−0.065	0.214	−0.001	0.991
M1 Macrophage	INOS (NOS2)	0.143	*	0.079	0.13	−0.064	0.148	−0.052	0.255	−0.056	0.257	−0.048	0.349	0.011	0.831	0.02	0.715
	IRF5	−0.081	0.102	−0.082	0.114	0.434	***	0.436	***	0.343	***	0.332	***	0.126	0.0154	0.108	0.045
	COX2 (PTGS2)	0.138	*	0.017	0.751	−0.005	0.907	−0.044	0.335	0.173	**	0.18	**	−0.096	0.0638	−0.004	0.94
M2 Macrophage	CD163	0.757	***	0.608	***	0.333	***	0.327	***	0.341	***	0.328	***	−0.126	0.0153	−0.049	0.368
	VSIG4	0.758	***	0.615	***	0.319	***	0.302	***	0.358	***	0.359	***	−0.085	0.102	0.002	0.972
	MS4A4A	0.748	***	0.598	***	0.315	***	0.318	***	0.35	***	0.335	***	−0.123	0.0176	−0.039	0.469
TAM	CD80	0.566	***	0.352	***	0.385	***	0.366	***	0.128	*	0.113	0.0282	−0.185	**	−0.134	0.0124
	CCL2	0.638	***	0.453	***	0.324	***	0.297	***	0.412	***	0.404	***	−0.003	0.959	0.108	0.045
	IL10	0.702	***	0.569	***	0.299	***	0.277	***	0.307	***	0.297	***	−0.156	*	−0.085	0.115
Monocyte	CD86	0.664	***	0.459	***	0.358	***	0.341	***	0.237	***	0.221	***	−0.18	**	−0.097	0.0729
	CD115 (CSF1R)	0.734	***	0.562	***	0.219	***	0.191	***	0.457	***	0.44	***	−0.112	0.0309	−0.02	0.705
NK cell	NCAM1	0.571	***	0.432	***	-0.37	***	-0.358	***	0.503	***	0.494	***	0.029	0.572	0.097	0.0728
	KIR2DL1	0.253	***	0.102	0.0498	0.084	0.0559	0.08	0.08	0.006	0.91	0.002	0.972	0.003	0.951	0.013	0.816
	KIR2DL3	0.348	***	0.167	*	0.191	***	0.183	***	−0.016	0.742	−0.047	0.363	0.007	0.894	0.044	0.414
	KIR2DL4	0.337	***	0.165	*	0.22	***	0.21	***	−0.112	0.0231	−0.135	*	−0.139	*	−0.093	0.0851
	KIR3DL1	0.252	***	0.133	0.0108	−0.016	0.712	−0.029	0.532	−0.004	0.941	−0.026	0.619	−0.002	0.971	0.056	0.301
	KIR3DL2	0.262	***	0.098	0.0597	0.147	**	0.14	*	0.073	0.135	0.062	0.226	−0.033	0.528	0.021	0.692
	KIR3DL3	0.093	0.0619	0.043	0.413	−0.014	0.746	−0.026	0.575	−0.098	0.0451	−0.096	0.0621	0.01	0.852	0.027	0.612
	KIR2DS4	0.286	***	0.153	*	0.169	**	0.162	**	−0.008	0.868	−0.034	0.504	0.005	0.919	−0.006	0.917
	CD94 (KLRD1)	0.525	***	0.311	***	0.295	***	0.285	***	0.159	*	0.149	*	−0.073	0.162	0.003	0.949
	CD314 (KLRK1)	0.442	***	0.211	***	−0.066	0.136	-0.039	0.396	0.212	***	0.196	**	−0.107	0.0393	−0.004	0.935
Neutrophil	CD66b (CEACAM8)	−0.014	0.779	0.002	0.969	0.014	0.752	0.002	0.973	−0.052	0.29	−0.034	0.51	−0.095	0.0663	−0.087	0.107
	CD11b (ITGAM)	0.714	***	0.542	***	0.316	***	0.296	***	0.418	***	0.415	***	−0.106	0.0406	−0.068	0.209
	CD15 (FUT4)	0.365	***	0.224	***	0.384	***	0.353	***	−0.097	0.0478	−0.109	0.0335	−0.023	0.652	0.024	0.659
	CCR7	0.065	0.188	−0.073	0.162	0.324	***	0.335	***	0.369	***	0.368	***	−0.072	0.169	0.038	0.487
	MPO	0.466	***	0.268	***	−0.015	0.738	−0.04	0.387	0.332	***	0.349	***	−0.024	0.647	0.043	0.424
Dendritic cell	CD1C	0.338	***	0.129	0.0131	0.198	***	0.183	***	0.389	***	0.387	***	−0.081	0.117	−0.026	0.626
	CD141	0.184	**	0.052	0.323	0.23	***	0.217	***	0.598	***	0.577	***	0.02	0.7	0.109	0.0432
	HLA-DPB1	0.603	***	0.375	***	0.505	***	0.496	***	0.221	***	0.21	***	−0.104	0.0457	−0.017	0.757
	HLA-DQB1	0.511	***	0.277	***	0.435	***	0.423	***	0.104	0.0345	0.083	0.105	−0.121	0.0202	−0.039	0.472
	HLA-DRA	0.531	***	0.295	***	0.472	***	0.458	***	0.107	0.0292	0.093	0.071	−0.098	0.0586	−0.009	0.869
	HLA-DPA1	0.557	***	0.344	***	0.453	***	0.443	***	0.156	*	0.144	*	−0.106	0.0404	−0.019	0.719
	BDCA-4 (NRP1)	0.536	***	0.436	***	0.314	***	0.324	***	0.535	***	0.524	***	0.149	*	0.173	*
	CD11c (ITGAX)	0.716	***	0.522	***	0.45	***	0.45	***	0.354	***	0.339	***	−0.151	*	−0.09	0.0937

None, correlation coefficient without adjustment; Purity, correlation adjusted by tumor purity; Cor, R value of Spearman’s correlation. *P<0.01. **P<0.001. ***P<0.0001.

**Figure 7:**
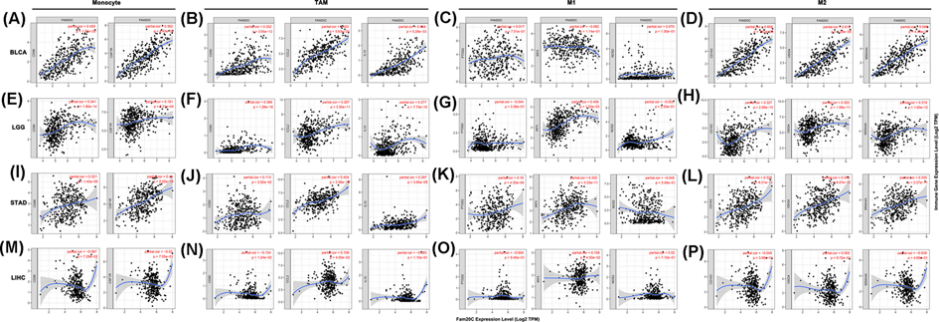
Fam20C expression correlated with macrophage polarization in BLCA, LGG, STAD, and LIHC Markers include CD86 and CSF1R of monocytes; CD80, CCL2, and IL10 of TAMs; PTGS2, IRF5, and NOS2 of M1 macrophages; and CD163, VSIG4, and MS4A4A of M2 macrophages. (**A**–**D**) Scatterplots of correlations between Fam20C expression and gene markers of monocytes (A), TAMs (B), M1 (C) and M2 macrophages (D) in BLCA (*n*=408). (**E**–**H**) Scatterplots of correlations between Fam20C expression and gene markers of monocytes (E), TAMs (F), M1 (G) and M2 macrophages (H) in LGG (*n*=516). (**I**–**L**) Scatterplots of correlations between Fam20C expression and gene markers of monocytes (I), TAMs (J), M1 (K) and M2 macrophages (L) in STAD (*n*=415). (**M**–**P**) The LISC was regarded as the control group showed that Fam20C expression has no significant correlation with macrophage polarization in LISC (*n*=371). The gene markers of monocytes (M), TAMs (N), M1 (O) and M2 macrophages (P).

In addition to the aforementioned overall changes, as shown in Figure 6, CD4^+^ T cells, macrophages, and dendritic cells, which were most closely related to Fam20C expression in BLCA, LGG, and STAD. However, with LISC, these three types were less significant. For the most expression markers levels of TAMs, monocytes, M2 macrophages had a robust association of Fam20C, specifically, CD80, CCL2, IL10, and Tim-3 of TAM, CD86 and CD115 of monocyte, CD163, VSIG4, and MS4A4A of M2 macrophage showed a strong association with Fam20C expression in BLCA, LGG, and STAD, despite no significant correlation of CD80 in STAD (*P*<0.0001; Figure 7A–P). To verify this finding, we performed the same analysis in GEPIA (Table 2). Consistently with TIMER, the results showed Fam20C may regulate macrophage polarization in BLCA, LGG, and STAD.

**Table 2:** Analysis of the correlation between Fam20C and gene markers of monocytes, TAMs, M1 macrophages, and M2 macrophages in GEPIA

Description	Gene markers	BLCA tumor		LGG tumor		STAD tumor		LIHC tumor	
		R	*P*	R	*P*	R	*P*	R	*P*
Monocyte	CD86	0.66	***	0.38	***	0.22	***	−0.16	*
	CD115	0.74	***	0.24	***	0.44	***	−0.078	0.13
TAM	CD80	0.59	***	0.41	***	0.12	0.012	−0.17	**
	CCL2	0.63	***	0.34	***	0.4	***	0.019	0.71
	IL10	0.69	***	0.29	***	0.31	***	−0.15	*
M1 macrophage	INOS (NOS2)	0.16	*	−0.044	0.31	−0.041	0.41	0.034	0.51
	IRF5	−0.056	0.26	0.46	***	0.34	***	0.14	*
	COX2 (PTGS2)	0.14	*	0.031	0.48	0.21	***	−0.055	0.29
M2 Macrophage	CD163	0.77	***	0.39	***	0.3	***	−0.13	0.012
	VSIG4	0.76	***	0.35	***	0.35	***	−0.067	0.2
	MS4A4A	0.75	***	0.35	***	0.35	***	−0.083	0.11

Tumor, correlation analysis in tumor tissue of TCGA. *P<0.01. **P<0.001. ***P<0.0001.

Significant correlation between key gene markers of the dendritic cells (CD1C, CD141, HLA-DPB1, HLA-DQB1, HLA-DRA, HLA-DPA1, BDCA-4, CD11C) and expression of Fam20C was observed in BLCA, LGG, and STAD compared with LIHC (Table 1). The results further supported a crucial role of Fam20C for DCs infiltration. With respect to Treg cells, Fam20C had a positive correlation with CD25, CCR8, FOXP3, CD127 in BLCA, LGG and STAD, despite no significant correlation of FOXP3 in LGG. Macrophages secrete a large number of chemokines such as CC-like chemokines CCL22 and CCL20, which induce Tregs to recruit to the tumor site, similarly DCs also induce Treg generation, and then promote the metastasis of cancer cells [47,48]. Whether Fam20C affects the DCs or macrophages and tumor metastasis need to be done for further studies.

In addition, a strong correlation existed between Fam20C and B cells, Tfh cells, Th9 cells, Th17 cells, and exhausted T cell markers. The relationships of Fam20C with CD8^+^ T cells, Th1 cells, Th2 cells, Th22 cells, neutrophils, and NK cells were partly different in BLCA, LGG, and STAD compared with LIHC. These observations, together with data from GEPIA, illustrate that Fam20C expression in BLCA, LGG, and STAD associates with different degree of immune cell infiltration in different way, further supporting Fam20C may be as an effective factor influencing patients survival and prognosis.

## Discussion

Fam20C is identified as Golgi casein kinase, which is expressed in a variety of tissues, including mineralized and non-mineralized tissues and body fluids [15,16,49]. Protein within Ser-X-Glu/pSer motif is phosphorylated by Fam20C in some 75% of human plasma and cerebrospinal fluid phosphoproteins. Focusing on the substrates of Fam20C, studies have shown that Fam20C not only regulates some biological processes, but also involved in tumor growth and metastasis [16]. Nevertheless, Fam20C has not been largely studied in the cancer field. It is now acknowledged that there is a relationship between Fam20C expression and tumor cell progression (mainly LUAD and BC) [23,24]. Therefore, it is desirable to speculate that the expression of Fam20C may affect the survival of patients through the progression of tumor cells. However, Fam20C expression in cancer and a consensus on the definition of other vital aspects like tumor cells metastasis are lacking. The role of Fam20C in cancer was observed in earlier studies but has not previously been dissected. Combined with previous research, our results remind that it should be noted that Fam20C may play diverse roles in various cancers. Reportedly, in Fam20C KO cells, the adhesion, migration, and invasion phenotype of BC cells could be rescued [16]. This might suggest that Fam20C is beneficial to the invasion and development of BC. However, in contrast to the situation with that previous study, we observed a relation between higher expression of Fam20C and a better prognosis in BRCA in PrognoScan database (data source from GEO) (Figure 2). More recently, in a trial conducted on the bioinformatics and human LUAD cells, hypoxia was indicated a poor prognostic factor for LUAD, and Fam20C was identified as a key gene associated with hypoxia in the progression of LUAD [24]. Consistently, LUAD expressed poorer prognosis in our research (Figure 2). In addition, we found that the expression of Fam20C was negatively correlated with tumor purity of LUAD, and positively correlated with immune cells infiltration, which further verified the relationship between Fam20C expression and poor prognosis (Supplementary Figure S3). A deeper understanding of these differences between previous studies using cancer cells and our study using the cohort of cancer patients may help develop a global view to generate cancer development mechanisms with Fam20C expression. Here, we present an integrated study on the Fam20C expression levels in pan-cancer, the association of Fam20C variations with prognosis among different cancers and the potential mechanisms underlying different clinicopathological features. Elevated Fam20C expression is associated with worse prognosis in BLCA, LGG, and STAD. Further, enhanced expression of Fam20C can affect lymph node metastasis with gastric cancer patients, indicating that Fam20C could be used as a predictor of tumor metastasis. Additionally, immune infiltration levels in BLCA, LGG, and STAD were positively correlated with Fam20C expression. Herein, the present study offers new insights into the clinical, prognostic, immunological understanding of Fam20C in different types of cancer.

In order to analyze the Fam20C expression levels among different cancer, we examined differential Fam20C expression across pan-cancer and their matched paracancer normal tissues of datasets from Oncomine and 32 cancer types of TCGA data from TIMER. Based on the Oncomine data showed that Fam20C had a higher expression level in brain and CNS, breast, cervical, colorectal, esophageal, head and neck, lymphoma, and pancreatic cancers. Further, in bladder, breast, colorectal and kidney cancer, a lower expression level of Fam20C was found (Figure 1A). However, given the data from TCGA in TIMER database, the results suggested Fam20C expression was relatively higher in HNSC, LIHC, LUAD, PRAD, and THCA than normal tissues while Fam20C expression descended in BLCA, BRCA, COAD, KICH, KIRP, LIHC, and SKCM (Figure 1B). Partial different results may be due to the difference in data sources, data collection approaches, and numbers of cancers in the study cohort. Nonetheless, in three separate databases, we found consistently poor prognostic value with Fam20C expression in BLCA, CESC, and brain cancers. Specifically, datasets of GEO were analyzed using PrognoScan showed that elevated Fam20C expression associated with worse prognosis in bladder, brain, colorectal, and lung cancers (Figure 2A,B,F–K). Further, applying TCGA database of GEPIA to the analysis showed higher mRNA levels of Fam20C had an increased risk for shorter time for OS and DFS in most tumor types, including BLCA, CESC, and brain cancer (GBM and LGG), HNSC, and UVM (Figure 4C–E,H–L,N). Kaplan–Meier Plotter explored elevated Fam20C expression associated with increased risk for RFS in CESC, and both OS and RFS in STAD (Figure 3E,W,X). Clinically, in gastric cancer patients with high expression levels of Fam20C correlated with increased risk for OS, FP, and PPS in stage 3–4, T2–T4, N0–N3, and M0 (Figure 5). These observations, together with clinicopathological features, illustrate that Fam20C is a newly identified multicancer-relevant gene with potential prognosis values in bladder, brain, cervical, and gastric cancer risk prediction, and supporting Fam20C might impact the patients with gastric cancer about lymph node metastasis.

Notably, another crucial part of the present study is that Fam20C expression is correlated with various immune infiltration levels in cancer, especially in BLCA, LGG, and STAD. We found a strong significant correlation between Fam20C and CD4^+^ T cells, macrophages, neutrophils, and DCs infiltration in BLCA, LGG, and STAD (Figure 6A,B,D), suggesting that Fam20C may influence both the extent of immune infiltration and the degree of activation of diverse immune cells. Moreover, the use of TIMER to estimate the degree of correlation between infiltrating immune cell markers and Fam20C expression is an attempt to identify the contributions of biomarkers. Recently, with the development of immune checkpoint inhibitors, biomarkers of immune cells can not only serve as prognostic markers, but also receive widespread attention as a new type of treatment [50]. More directly, we found the association between Fam20C and immune cells markers suggested Fam20C might regulate tumor immunology in BLCA, LGG, and STAD. Among this, genetic markers of M1 macrophages, for example INOS and COX2 indicated no significant correlation with Fam20C expression, while M2 macrophage gene markers such as CD163, VSIG4, and MS4A4A exhibited high correlations (Table 1). These findings suggest the potential regulatory role of Fam20C in polarization of TAMs. TAM can promote tumor growth by suppressing immune clearance, promoting tumor cell proliferation, and stimulating angiogenesis [51]. We have also identified Fam20C might have the potential to activate Treg cells and induced T-cell exhaustion. Most these markers were positively correlated with Fam20C, including CD25, CCR8, FOXP3, CD127, PD-1, Tim-3, CTLA4, LAG3, and GZMB (Table 1). As noted in previous studies, Tregs are often associated with a poor clinical outcome, their ability to promote progression of cancer through limiting antitumor immunity and promoting angiogenesis [52]. Exhausted T cells are defined as a group of T cells with poor effector function and sustained expression of inhibitory receptors [53]. During tumor immunity, CD4^+^ T cells and CD8^+^ T cells exhaustion will promote tumorigenesis and development, in this process PD-1 is the major inhibitory receptor [54]. Also, PD-1 showed a strong association with Fam20C expression in BLCA, LGG, and STAD. In addition, we observed in BLCA, LGG, and STAD there was a high correlation between Fam20C and the markers of T helper cells (Th1, Th2, Th9, Tfh, and Th17). These findings imply an alternative mechanism for Fam20C regulated activation of T cells. Here we show major correlations between CD4^+^ T cells, neutrophils, DCs, M2 macrophages, TAMs, Tregs, exhausted T cells, and T helper cells with Fam20C, supporting the the important role of Fam20C in the immune contexture in BLCA, LGG, and STAD.

It is still unclear that what role Fam20C expression plays in the process of tumorigenesisor in pan-cancer. More recently, some studies have presented possible mechanisms of Fam20C expression correlates with poor prognosis. For the tumor environment, it is axiomatic that hypoxia is a common feature of cancers [55,56]. DNA methylation plays important regulatory roles in cancer progression. An analysis of DNA methylation profiles of 533 LUAD patients showed FAM20C was identified as one of hypoxia-related key genes, specifically, hypoxia in LUAD cells inhibited DNA methylation of Fam20C gene, promoted Fam20C gene expression, and further led to deterioration of LUAD [24]. Another evidence of supporting the role of Fam20C in tumor migration, revealed that the Fam20C inhibitor (FL-1607) designed by structure-based molecular modeling had the effects of antitumor growth, inducing cell apoptosis and inhibiting cell migration [23]. Together with our finding that Fam20C impacted the prognosis in gastric cancer patients with lymph node metastasis, further provide an evidence about Fam20C expression might influence cancer cells migration. Recent studies have found that EMT (epithelial–mesenchymal transition) is closely related to the occurrence of multiple cancers and the proliferation, migration, and invasion of cancer cells [57]. And CDH2 (cadherin-2), one of the markers of EMT, was founded in the Fam20C phosphoproteome [16]. As predict, E-cadherin (CDH1) converted into CDH2 negatively correlated with Fam20C expression, other markers of EMT, including CDH2, SNAIL, TWIST, ZEB1 and ZEB2 were positively correlated with the expression of Fam20C (Supplementary Table S2). This likely indicates Fam20C participates in the EMT process. Therefore, enhancement of cancer cells in adhesion and migration, which may be accompanied by EMT, could be an underlying regulatory mechanism associated with Fam20C and bad prognosis.

Our study showed that increased expression of Fam20C is linked to poor prognosis in multiple cancer types, and the infiltrating immune cells associated with Fam20C expression in TME. These findings may allow better prognostic prediction and providing immuno-oncological perspective of regarding Fam20C as a prognostic marker. Nevertheless, even if we collected the information among multiple different databases, this research still had restrictions. Initially, a large amount of sequencing and microarray data were gathered and analyzed for tumor tissues, which ignored the heterogeneity of cells in the tumor tissue, so there was a certain systematic bias. Further, the applications of single-cell sequencing can provide high-resolution research to solve this problem. Second, due to the contradictory findings of individual cancers in different databases, we cannot determine the prognostic value of Fam20C in these cancers. Third, our research only performed a bioinformatics analysis of Fam20C and patient survival value in multiple databases, but did not perform *in vivo/in vitro* experiments to verify. Next, we will complement *in vivo/in vitro* experiments to achieve the mechanisms of Fam20C in different cancer types at the cellular and molecular levels. Fourth, although Fam20C expression was found to be associated with immune cell infiltration and patient’s survival, it has not been demonstrated that Fam20C affects patient’s survival through immune infiltration. Whether the expression or function of Fam20C and their products affects cancer cell growth and migration in the clinical setting is an important topic for future studies.

In summary, elevated Fam20C expression can impact prognostic value in pan-cancer and increase degree of immune infiltration. In BLCA, LGG and STAD, Fam20C expression potentially contributes to the polarization of TAM, activation of Treg cells and T helper cells, and induction of T cell exhaustion. Therefore, Fam20C might be a prognostic biomarker in pan-cancer and its expression is in association with immune infiltration in BLCA, LGG, and STAD.

## Supplementary Material

Supplementary Figures S1-S3 and Table S1-S2

## Data Availability

Our study used public online database. The data can be accessed by following websites: https://www.oncomine.org/, http://dna00.bio.kyutech.ac.jp/PrognoScan/index.html, https://kmplot.com/analysis/, http://gepia.cancer-pku.cn/index.html, https://cistrome.shinyapps.io/timer/.

## Abbreviations

ATP: adenosine triphosphate
BC: breast cancer
BLCA: bladder urothelial carcinoma
BRCA: breast invasive carcinoma
CCL2: C-C motif chemokine ligand 2
CDH2: cadherin-2
CD80: CD80 molecule
CESC: cervical squamous cell carcinoma
CI: confidence interval
CNS: central nervous system
COAD: colon adenocarcinoma
COX2: cytochrome c oxidase II
DFS: disease-free survival
DLBC: diffuse large B-cell lymphoma
DMFS: distant metastasis-free survival
DSS: disease-specific survival
EFS: event-free survival
EMT: epithelial–mesenchymal transition
Fam20C: family with sequence similarity 20-member C
FP: first progression
GBM: glioblastoma multiforme
GEO: Gene Expression Omnibus
GEPIA: Gene Expression Profiling Interactive Analysis
HNSC: head and neck squamous carcinoma
HR: hazard ratio
INOS: inducible nitricoxide synthase
KICH: kidney chromophobe
KIRP: kidney renal papillary cell carcinoma
LGG: brain lower grade glioma
LIHC: liver hepatocellular carcinoma
LUAD: lung adenocarcinoma
NK: natural killer
OS: overall survival
PD-1: programmed cell death protein 1
PPS: post-progression survival
PRAD: prostate adenocarcinoma
RFS: recurrence/relapse-free survival
SKCM: skin cutaneous melanoma
STAD: stomach adenocarcinoma
TAM: tumor-associated macrophage
TCGA: The Cancer Genome Atlas
Tfh: follicular helper T cell
THCA: thyroid carcinoma
Th1/2/9/17/22: T-helper 1/2/9/17/22 cell
TIIC: tumor-infiltrating immune cell
TIL: tumor-infiltrating lymphocyte
TIMER: Tumor Immune Estimation Resource
Tim-3: T-cell immunoglobulin mucin 3
TME: tumor microenvironment
TPM: transcripts per million
TWIST: twist family bHLH transcription factor
UVM: uveal melanoma
ZEB1/2: zinc finger E-box-binding protein 1/2
