# Supplementary material for: Prognostic and immunological role of Fam20C in pan-cancer
Source: Biosci Rep. 2021 Jan 5;41(1):BSR20201920. doi: 10.1042/BSR20201920 (PMC7786334; doi:10.1042/BSR20201920)
Supplement: Supplementary Figures S1-S3 and Table S1-S2 [file BSR-2020-1920_supp.pdf]

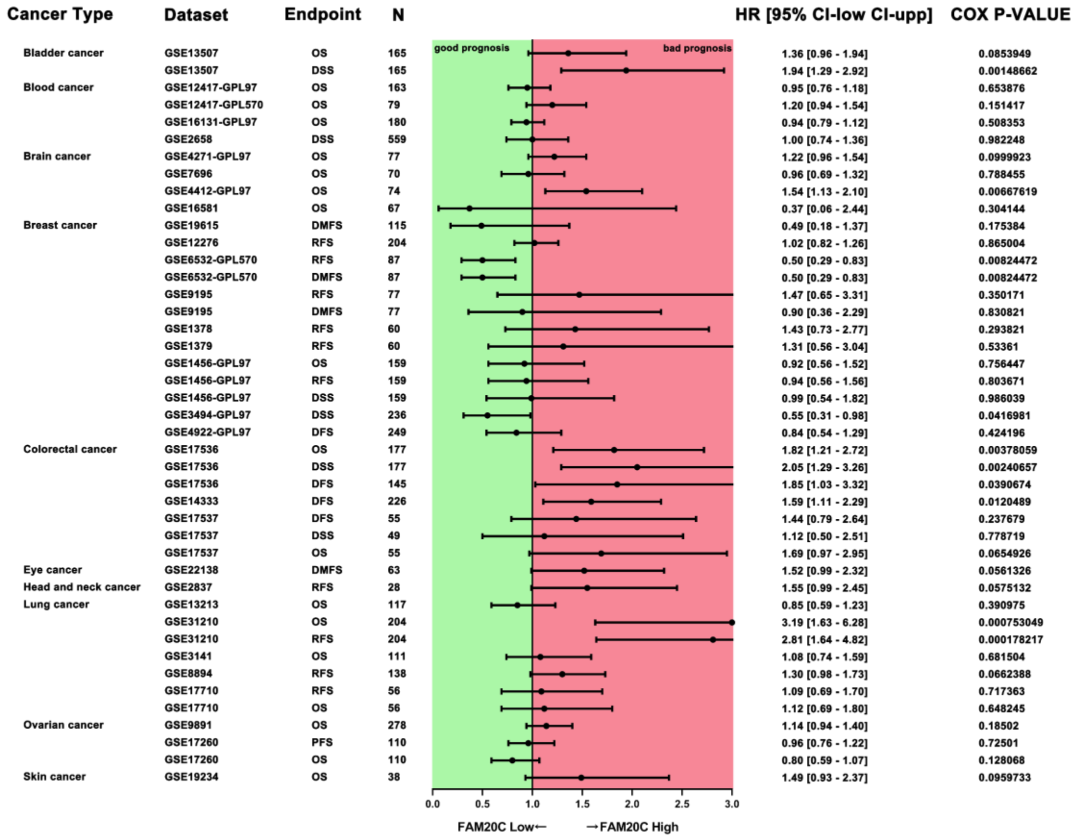

Supplementary Figure1 Correlation of Fam20C mRNA expression and clinical outcome in different cancer types. Green area represent good prognosis, in contrast, red area represent bad prognosis. Black squares represent hazard ratio. Short bars appear due to limited sample size for parameters. OS, overall survival; PFS, progression free survival; DSS, disease specific survival; DMFS, distant metastasis free survival; RFS, relapse free survival; *$P < 0.05$.

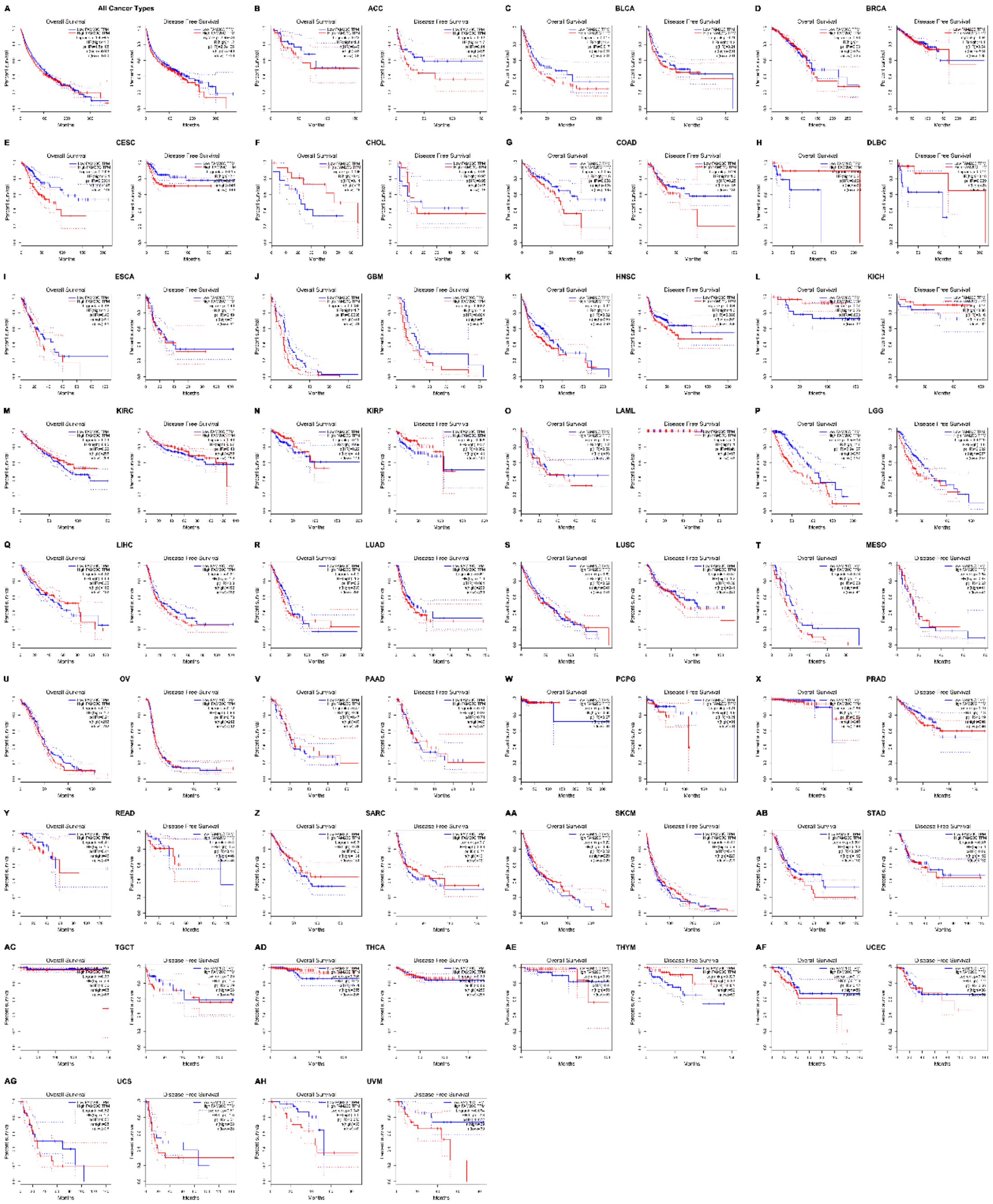

Supplementary Figure 2. Correlation of Fam20C expression with prognostic values in cancers. Overall survival and disease free survival of **(A)** all cancer types **(B)** ACC, Adrenocortical carcinoma **(C)** BLCA, Bladder Urothelial Carcinoma **(D)** BRCA, Breast invasive carcinoma **(E)** CESC, Cervical squamous cell carcinoma and endocervical adenocarcinoma **(F)** CHOL, Cholangio carcinoma **(G)** COAD, Colon adenocarcinoma **(H)** DLBC, Lymphoid Neoplasm Diffuse Large B-cell Lymphoma **(I)** ESCA, Esophageal carcinoma **(J)** GBM, Glioblastoma multiforme **(K)** HNSC, Head and Neck squamous cell carcinoma **(L)** KICH, Kidney Chromophobe **(M)** KIRC, Kidney renal clear cell carcinoma **(N)** KIRP, Kidney renal papillary cell carcinoma **(O)** LAML, Acute Myeloid Leukemia **(P)** LGG, Brain Lower Grade Glioma **(Q)** LIHC, Liver hepatocellular carcinoma **(R)** LUAD, Lung adenocarcinoma **(S)** LUSC, Lung squamous cell carcinoma **(T)** MESO, Mesothelioma **(U)** OV, Ovarian serous cystadenocarcinoma **(V)** PAAD, Pancreatic adenocarcinoma **(W)** PCPG, Pheochromocytoma and Paraganglioma **(X)** PRAD, Prostate adenocarcinoma **(Y)** READ, Rectum

adenocarcinoma **(Z)** SARC, Sarcoma **(AA)** SKCM, Skin Cutaneous Melanoma **(AB)** STAD, Stomach adenocarcinoma **(AC)** TGCT, Testicular Germ Cell Tumors **(AD)** THCA, Thyroid carcinoma **(AE)** THYM, Thymoma **(AF)** UCEC, Uterine Corpus Endometrial Carcinoma **(AG)** UCS, Uterine Carcinosarcoma and **(AH)** UVM, Uveal Melanoma.

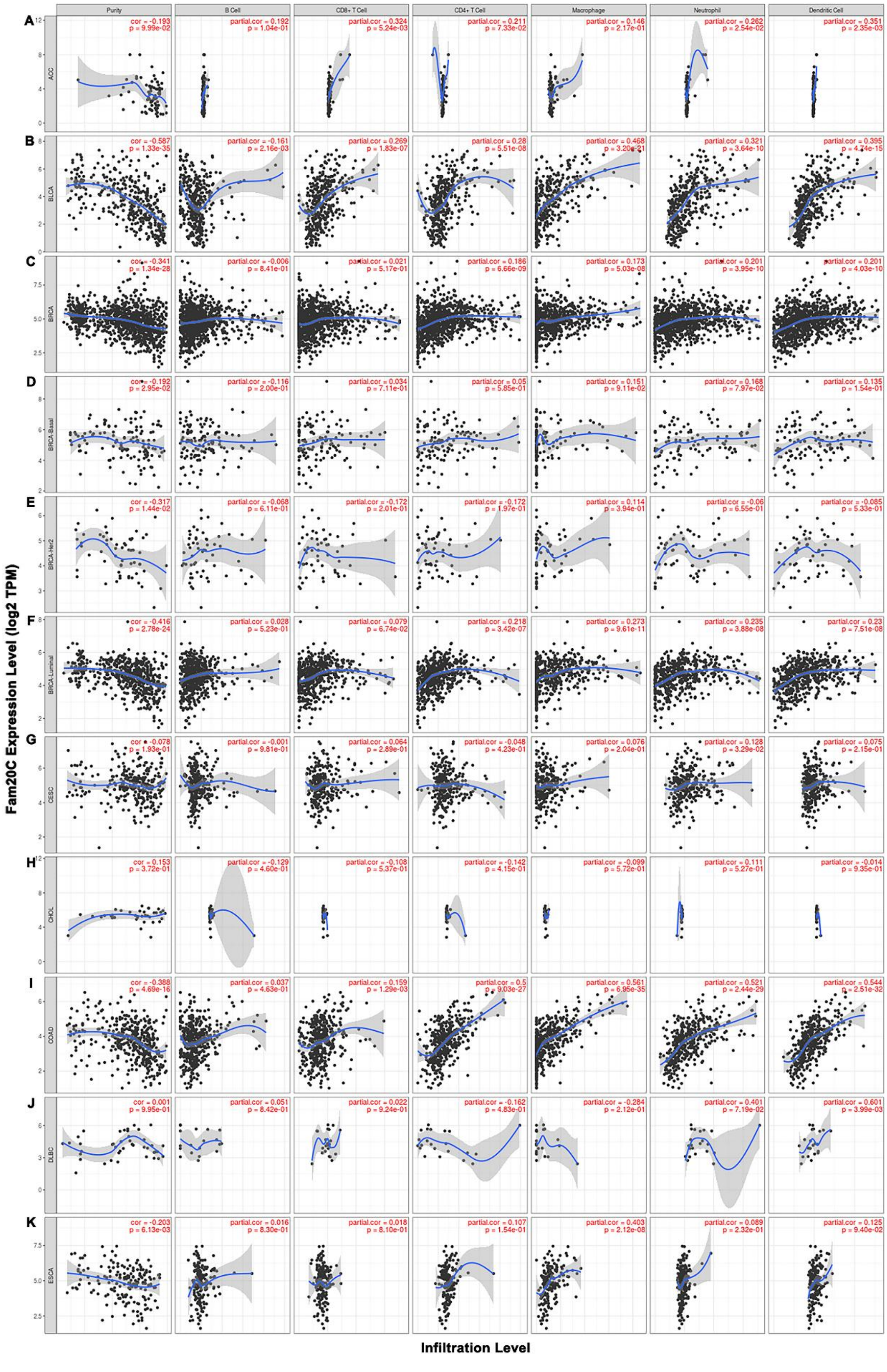
Purity
B Cell
CD8+ T Cell
CD4+ T Cell
Macrophage
Neutrophil
Dendritic Cell
A
ACC
cor = -0.193 p = 9.99e-02
partial.cor = 0.192 p = 1.04e-01
partial.cor = 0.324 p = 5.24e-03
partial.cor = 0.211 p = 7.33e-02
partial.cor = 0.146 p = 2.17e-01
partial.cor = 0.262 p = 2.54e-02
partial.cor = 0.351 p = 2.35e-03
B
BLCA
cor = -0.587 p = 1.33e-35
partial.cor = -0.161 p = 2.16e-03
partial.cor = 0.269 p = 1.83e-07
partial.cor = 0.28 p = 5.51e-08
partial.cor = 0.468 p = 3.20e-21
partial.cor = 0.321 p = 3.64e-10
partial.cor = 0.395 p = 4.74e-15
C
BRCA
cor = -0.341 p = 1.34e-28
partial.cor = -0.006 p = 8.41e-01
partial.cor = 0.021 p = 5.17e-01
partial.cor = 0.186 p = 6.66e-09
partial.cor = 0.173 p = 5.03e-08
partial.cor = 0.201 p = 3.95e-10
partial.cor = 0.201 p = 4.03e-10
D
BRCA-Basal
cor = -0.192 p = 2.95e-02
partial.cor = -0.116 p = 2.00e-01
partial.cor = 0.034 p = 7.11e-01
partial.cor = 0.05 p = 5.85e-01
partial.cor = 0.151 p = 9.11e-02
partial.cor = 0.168 p = 7.97e-02
partial.cor = 0.135 p = 1.54e-01
E
BRCA-Her2
cor = -0.317 p = 1.44e-02
partial.cor = -0.068 p = 6.11e-01
partial.cor = -0.172 p = 2.01e-01
partial.cor = -0.172 p = 1.97e-01
partial.cor = 0.114 p = 3.94e-01
partial.cor = -0.06 p = 6.55e-01
partial.cor = -0.085 p = 5.33e-01
F
BRCA-Luminal
cor = -0.416 p = 2.78e-24
partial.cor = 0.028 p = 5.23e-01
partial.cor = 0.079 p = 6.74e-02
partial.cor = 0.218 p = 3.42e-07
partial.cor = 0.273 p = 9.61e-11
partial.cor = 0.235 p = 3.88e-08
partial.cor = 0.23 p = 7.51e-08
G
CESC
cor = -0.078 p = 1.93e-01
partial.cor = -0.001 p = 9.81e-01
partial.cor = 0.064 p = 2.89e-01
partial.cor = -0.048 p = 4.23e-01
partial.cor = 0.076 p = 2.04e-01
partial.cor = 0.128 p = 3.29e-02
partial.cor = 0.075 p = 2.15e-01
H
CHOL
cor = 0.153 p = 3.72e-01
partial.cor = -0.129 p = 4.60e-01
partial.cor = -0.108 p = 5.37e-01
partial.cor = -0.142 p = 4.15e-01
partial.cor = -0.099 p = 5.72e-01
partial.cor = 0.111 p = 5.27e-01
partial.cor = -0.014 p = 9.35e-01
I
COAD
cor = -0.388 p = 4.69e-16
partial.cor = 0.037 p = 4.63e-01
partial.cor = 0.159 p = 1.29e-03
partial.cor = 0.5 p = 9.03e-27
partial.cor = 0.561 p = 6.95e-35
partial.cor = 0.521 p = 2.44e-29
partial.cor = 0.544 p = 2.51e-32
J
DLBC
cor = 0.001 p = 9.95e-01
partial.cor = 0.051 p = 8.42e-01
partial.cor = 0.022 p = 9.24e-01
partial.cor = -0.162 p = 4.83e-01
partial.cor = -0.284 p = 2.12e-01
partial.cor = 0.401 p = 7.19e-02
partial.cor = 0.601 p = 3.99e-03
K
ESCA
cor = -0.203 p = 6.13e-03
partial.cor = 0.016 p = 8.30e-01
partial.cor = 0.018 p = 8.10e-01
partial.cor = 0.107 p = 1.54e-01
partial.cor = 0.403 p = 2.12e-08
partial.cor = 0.089 p = 2.32e-01
partial.cor = 0.125 p = 9.40e-02
Fam20C Expression Level (log2 TPM)
Infiltration Level

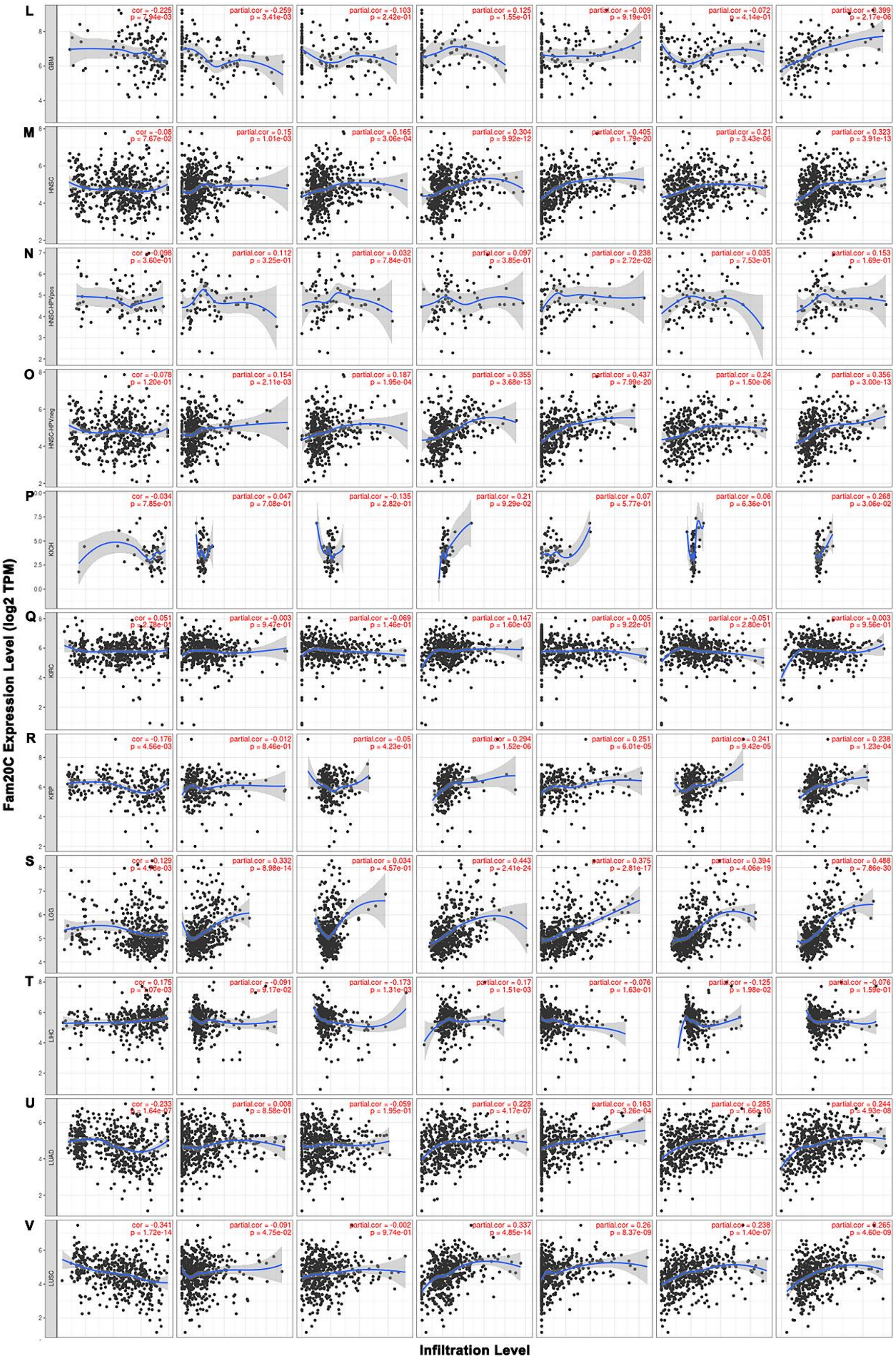
Fam20C Expression Level (log2 TPM)
Infiltration Level
L
GBM
cor = -0.225
p = 7.94e-03
partial.cor = -0.259
p = 3.41e-03
partial.cor = -0.103
p = 2.42e-01
partial.cor = 0.125
p = 1.55e-01
partial.cor = -0.009
p = 9.19e-01
partial.cor = -0.072
p = 4.14e-01
partial.cor = 0.399
p = 2.17e-06
M
HNSC
cor = -0.08
p = 7.67e-02
partial.cor = 0.15
p = 1.01e-03
partial.cor = 0.165
p = 3.06e-04
partial.cor = 0.304
p = 9.92e-12
partial.cor = 0.405
p = 1.79e-20
partial.cor = 0.21
p = 3.43e-06
partial.cor = 0.323
p = 3.91e-13
N
HNSC-HPVpos
cor = -0.098
p = 3.60e-01
partial.cor = 0.112
p = 3.25e-01
partial.cor = 0.032
p = 7.84e-01
partial.cor = 0.097
p = 3.85e-01
partial.cor = 0.238
p = 2.72e-02
partial.cor = 0.035
p = 7.53e-01
partial.cor = 0.153
p = 1.69e-01
O
HNSC-HPVneg
cor = -0.078
p = 1.20e-01
partial.cor = 0.154
p = 2.11e-03
partial.cor = 0.187
p = 1.95e-04
partial.cor = 0.355
p = 3.68e-13
partial.cor = 0.437
p = 7.99e-20
partial.cor = 0.24
p = 1.50e-06
partial.cor = 0.356
p = 3.00e-13
P
KICH
cor = -0.034
p = 7.85e-01
partial.cor = 0.047
p = 7.08e-01
partial.cor = -0.135
p = 2.82e-01
partial.cor = 0.21
p = 9.29e-02
partial.cor = 0.07
p = 5.77e-01
partial.cor = 0.06
p = 6.36e-01
partial.cor = 0.268
p = 3.06e-02
Q
KIRC
cor = 0.051
p = 2.78e-01
partial.cor = -0.003
p = 9.47e-01
partial.cor = -0.069
p = 1.46e-01
partial.cor = 0.147
p = 1.60e-03
partial.cor = 0.005
p = 9.22e-01
partial.cor = -0.051
p = 2.80e-01
partial.cor = 0.003
p = 9.56e-01
R
KIRP
cor = -0.176
p = 4.56e-03
partial.cor = -0.012
p = 8.46e-01
partial.cor = -0.05
p = 4.23e-01
partial.cor = 0.294
p = 1.52e-06
partial.cor = 0.251
p = 6.01e-05
partial.cor = 0.241
p = 9.42e-05
partial.cor = 0.238
p = 1.23e-04
S
LGG
cor = -0.129
p = 4.98e-03
partial.cor = 0.332
p = 8.98e-14
partial.cor = 0.034
p = 4.57e-01
partial.cor = 0.443
p = 2.41e-24
partial.cor = 0.375
p = 2.81e-17
partial.cor = 0.394
p = 4.06e-19
partial.cor = 0.488
p = 7.86e-30
T
LIHC
cor = 0.175
p = 1.07e-03
partial.cor = -0.091
p = 9.17e-02
partial.cor = -0.173
p = 1.31e-03
partial.cor = 0.17
p = 1.51e-03
partial.cor = -0.076
p = 1.63e-01
partial.cor = -0.125
p = 1.98e-02
partial.cor = -0.076
p = 1.59e-01
U
LUAD
cor = -0.233
p = 1.64e-07
partial.cor = 0.008
p = 8.58e-01
partial.cor = -0.059
p = 1.95e-01
partial.cor = 0.228
p = 4.17e-07
partial.cor = 0.163
p = 3.26e-04
partial.cor = 0.285
p = 1.68e-10
partial.cor = 0.244
p = 4.93e-08
V
LUSC
cor = -0.341
p = 1.72e-14
partial.cor = -0.091
p = 4.75e-02
partial.cor = -0.002
p = 9.74e-01
partial.cor = 0.337
p = 4.85e-14
partial.cor = 0.26
p = 8.37e-09
partial.cor = 0.238
p = 1.40e-07
partial.cor = 0.265
p = 4.60e-09

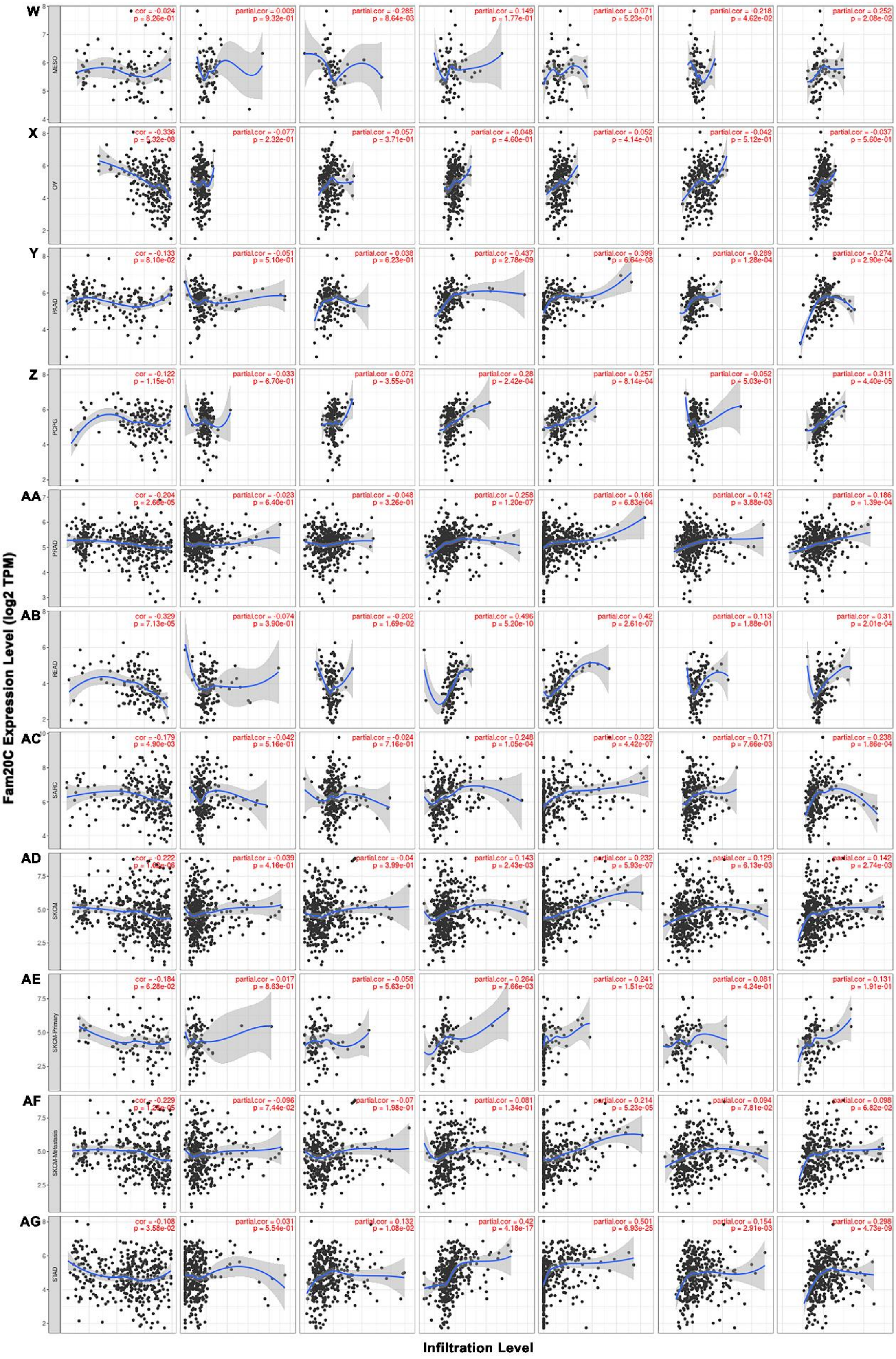

Fam20C Expression Level (log2 TPM)
Infiltration Level
W
MESO
cor = -0.024 p = 8.26e-01
partial.cor = 0.009 p = 9.32e-01
partial.cor = -0.285 p = 8.64e-03
partial.cor = 0.149 p = 1.77e-01
partial.cor = 0.071 p = 5.23e-01
partial.cor = -0.218 p = 4.62e-02
partial.cor = 0.252 p = 2.08e-02
X
OV
cor = -0.336 p = 5.32e-08
partial.cor = -0.077 p = 2.32e-01
partial.cor = -0.057 p = 3.71e-01
partial.cor = -0.048 p = 4.60e-01
partial.cor = 0.052 p = 4.14e-01
partial.cor = -0.042 p = 5.12e-01
partial.cor = -0.037 p = 5.60e-01
Y
PAAD
cor = -0.133 p = 8.10e-02
partial.cor = -0.051 p = 5.10e-01
partial.cor = 0.038 p = 6.23e-01
partial.cor = 0.437 p = 2.78e-09
partial.cor = 0.399 p = 6.64e-08
partial.cor = 0.289 p = 1.28e-04
partial.cor = 0.274 p = 2.90e-04
Z
PCPG
cor = -0.122 p = 1.15e-01
partial.cor = -0.033 p = 6.70e-01
partial.cor = 0.072 p = 3.55e-01
partial.cor = 0.28 p = 2.42e-04
partial.cor = 0.257 p = 8.14e-04
partial.cor = -0.052 p = 5.03e-01
partial.cor = 0.311 p = 4.40e-05
AA
PRAD
cor = -0.204 p = 2.66e-05
partial.cor = -0.023 p = 6.40e-01
partial.cor = -0.048 p = 3.26e-01
partial.cor = 0.258 p = 1.20e-07
partial.cor = 0.166 p = 6.83e-04
partial.cor = 0.142 p = 3.88e-03
partial.cor = 0.186 p = 1.39e-04
AB
READ
cor = -0.329 p = 7.13e-05
partial.cor = -0.074 p = 3.90e-01
partial.cor = -0.202 p = 1.69e-02
partial.cor = 0.496 p = 5.20e-10
partial.cor = 0.42 p = 2.61e-07
partial.cor = 0.113 p = 1.88e-01
partial.cor = 0.31 p = 2.01e-04
AC
SARC
cor = -0.179 p = 4.90e-03
partial.cor = -0.042 p = 5.16e-01
partial.cor = -0.024 p = 7.16e-01
partial.cor = 0.248 p = 1.05e-04
partial.cor = 0.322 p = 4.42e-07
partial.cor = 0.171 p = 7.66e-03
partial.cor = 0.238 p = 1.86e-04
AD
SKCM
cor = -0.222
partial.cor = -0.039 p = 4.16e-01
partial.cor = -0.04 p = 3.99e-01
partial.cor = 0.143 p = 2.43e-03
partial.cor = 0.232 p = 5.93e-07
partial.cor = 0.129 p = 6.13e-03
partial.cor = 0.142 p = 2.74e-03
AE
SKCM-Primary
cor = -0.184 p = 6.28e-02
partial.cor = 0.017 p = 8.63e-01
partial.cor = -0.058 p = 5.63e-01
partial.cor = 0.264 p = 7.66e-03
partial.cor = 0.241 p = 1.51e-02
partial.cor = 0.081 p = 4.24e-01
partial.cor = 0.131 p = 1.91e-01
AF
SKCM-Metastasis
cor = -0.229
partial.cor = -0.096 p = 7.44e-02
partial.cor = -0.07 p = 1.98e-01
partial.cor = 0.081 p = 1.34e-01
partial.cor = 0.214 p = 5.23e-05
partial.cor = 0.094 p = 7.81e-02
partial.cor = 0.098 p = 6.82e-02
AG
STAD
cor = -0.108 p = 3.58e-02
partial.cor = 0.031 p = 5.54e-01
partial.cor = 0.132 p = 1.08e-02
partial.cor = 0.42 p = 4.18e-17
partial.cor = 0.501 p = 6.93e-25
partial.cor = 0.154 p = 2.91e-03
partial.cor = 0.298 p = 4.73e-09

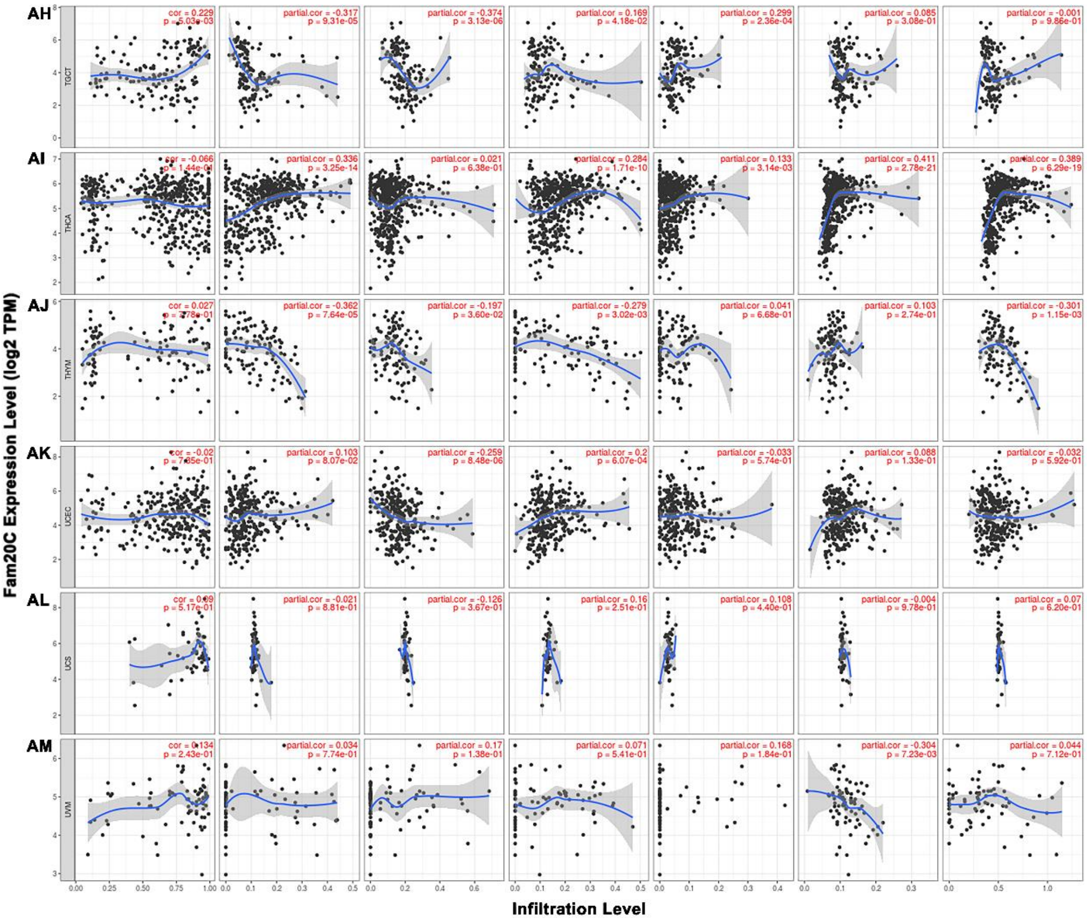

Supplementary Figure 3. Correlation of Fam20C expression with immune infiltration level across different cancer types cancers via TIMER database. **(A)** ACC, Adrenocortical carcinoma **(B)** BLCA, Bladder Urothelial Carcinoma **(C)** BRCA, Breast invasive carcinoma **(D)** BRCA-Basal, Breast invasive carcinoma-Basal **(E)** BRCA-Her2, Breast invasive carcinoma-Her2 **(F)** BRCA-Luminal, Breast invasive carcinoma-Luminal **(G)** CESC, Cervical squamous cell carcinoma and endocervical adenocarcinoma **(H)** CHOL, Cholangio carcinoma **(I)** COAD, Colon adenocarcinoma **(J)** DLBC, Lymphoid Neoplasm Diffuse Large B-cell Lymphoma **(K)** ESCA, Esophageal carcinoma **(L)** GBM, Glioblastoma multiforme **(M)** HNSC, Head and Neck squamous cell carcinoma **(N)** HNSC-HPVpos, Head and Neck squamous cell carcinoma-HPVpos **(O)** HNSC-HPVneg, Head and Neck squamous cell carcinoma-HPVneg **(P)** KICH, Kidney Chromophobe **(Q)** KIRC, Kidney renal clear cell carcinoma **(R)** KIRP, Kidney renal papillary cell carcinoma **(S)** LGG, Brain Lower Grade Glioma **(T)** LIHC, Liver hepatocellular carcinoma **(U)** LUAD, Lung adenocarcinoma **(V)** LUSC, Lung squamous cell carcinoma **(W)** MESO, Mesothelioma **(X)** OV, Ovarian serous cystadenocarcinoma **(Y)** PAAD, Pancreatic adenocarcinoma **(Z)** PCPG, Pheochromocytoma and Paraganglioma **(AA)** PRAD, Prostate adenocarcinoma **(AB)** READ, Rectum adenocarcinoma **(AC)** SARC, Sarcoma **(AD)** SKCM, Skin Cutaneous Melanoma **(AE)** SKCM-Primary, Skin Cutaneous Melanoma-Primary **(AF)** SKCM-Metastasis, Skin Cutaneous Melanoma-Metastasis **(AG)** STAD, Stomach adenocarcinoma **(AH)** TGCT, Testicular Germ Cell Tumors **(AI)** THCA, Thyroid carcinoma **(AJ)** THYM, Thymoma **(AK)** UCEC, Uterine Corpus Endometrial Carcinoma **(AL)** UCS, Uterine Carcinosarcoma and **(AM)** UVM, Uveal Melanoma.

Supplementary Table

Supplementary Table 1. Fam20C expression in cancer versus normal tissue in ONCOMINE.

Cancer Site	Cancer Type	P Value	Sample Size	Fold Change	Up/Down Regulated Gene Rank	Reference (PMID)
Bladder	Superficial Bladder Cancer	3.13E-17	126	-2.777	114(1%)	20421545
Brain and CNS	Glioblastoma	2.29E-15	81	5.225	471(3%)	16616334
	Glioblastoma	4.16E-4	22	3.109	1087(6%)	16697959
	Glioblastoma	9.92E-6	80	3.265	1168(6%)	18565887
Breast	Invasive Breast Carcinoma Stroma	9.10E-17	53	2.662	662(4%)	18438415
	Ductal Breast Carcinoma in Situ Epithelia	1.02E-5	9	-2.586	221(2%)	19187537
	Invasive Breast Carcinoma Stroma	1.53E-22	53	-8.363	888(5%)	18438415
Cervical	Cervical Cancer	4.16E-14	20	3.101	20(1%)	17510386
	Cervical Squamous Cell Carcinoma	1.39E-8	40	3.218	535(3%)	18191186
Colorectal	Rectal Adenocarcinoma	1.04E-15	65	2.146	1692(9%)	20725992
	Colon Adenoma Epithelia	8.02E-5	5	-2.064	1346(7%)	20957034
Esophageal	Esophageal Adenocarcinoma	1.46E-10	75	2.044	936(5%)	21152079
Head and Neck	Tongue Carcinoma	2.71E-6	15	2.248	475(3%)	17510386
	Oropharyngeal Carcinoma	9.78E-5	6	2.441	598(4%)	17510386
Kidney	Renal Oncocytoma	4.76E-7	4	-8.861	4(1%)	19445733
Lymphoma	Diffuse Large B-Cell Lymphoma	2.50E-14	44	3.234	892(5%)	19412164
	Unspecified Peripheral T-Cell Lymphoma	3.81E-11	28	2.356	895(5%)	17304354
Other	Skin Basal Cell Carcinoma	3.30E-6	15	5.769	268(2%)	18442402
	Teratoma, NOS	3.39E-6	14	2.991	1567(9%)	16424014
Pancreatic	Pancreatic Ductal Adenocarcinoma	2.00E-12	39	2.294	286(2%)	19260470

Supplementary Table

Supplementary Table 2. Analysis of the correlation between Fam20C and gene markers of epithelial-mesenchymal transition (EMT) in TIMER.

Marker	BLCA				STAD				LIHC			
	None		Purity		None		Purity		None		Purity	
	Cor	P	Cor	P	Cor	P	Cor	P	Cor	P	Cor	P
CDH1	-0.248	***	-0.216	***	-0.192	***	-0.168	*	-0.043	0.407	-0.044	0.412
CDH2	0.579	***	0.498	***	0.433	***	0.441	***	0.069	0.185	0.027	0.613
SNAIL	0.69	***	0.525	***	0.313	***	0.301	***	-0.056	0.279	0.039	0.465
TWIST	0.694	***	0.58	***	0.42	***	0.416	***	-0.052	0.314	0.019	0.725
ZEB1	0.649	***	0.536	***	0.647	***	0.637	***	0.07	0.18	0.043	0.427
ZEB2	0.779	***	0.652	***	0.576	***	0.562	***	-0.072	0.166	0.016	0.765
